# LncRNA-mRNA Co-Expression Analysis Identifies AL133346.1/CCN2 as Biomarkers in Pediatric B-Cell Acute Lymphoblastic Leukemia

**DOI:** 10.3390/cancers12123803

**Published:** 2020-12-17

**Authors:** Marta Cuadros, Daniel J. García, Alvaro Andrades, Alberto M. Arenas, Isabel F. Coira, Carlos Baliñas-Gavira, Paola Peinado, María I. Rodríguez, Juan Carlos Álvarez-Pérez, Francisco Ruiz-Cabello, Mireia Camós, Antonio Jiménez-Velasco, Pedro P. Medina

**Affiliations:** 1 Department of Biochemistry and Molecular Biology III and Immunology, University of Granada, Av. de la Investigación 11, 18016 Granada, Spain; mcuadros@ugr.es (M.C.); djgargar@ugr.es (D.J.G.); maria.rodriguez@genyo.es (M.I.R.); fruizc@ugr.es (F.R.-C.); 2 GENYO, Centre for Genomics and Oncological Research, Pfizer/University of Granada/Andalusian Regional Government, Av. de la Ilustración 114, 18016 Granada, Spain; alande@ugr.es (A.A.); amam@ugr.es (A.M.A.); isabel.fernandezcoira@unige.ch (I.F.C.); carlos.balinas@genyo.es (C.B.-G.); paola.peinado@genyo.es (P.P.); carlosalvarez@ugr.es (J.C.Á.-P.); 3 Instituto de Investigación Biosanitaria (ibs. Granada), Av. Fuerzas Armadas 2, 18014 Granada, Spain; 4 Department of Biochemistry and Molecular Biology I, University of Granada, Av. de Fuente Nueva S/N, 18071 Granada, Spain; 5 Department of Clinical Analysis and Immunology, UGC Laboratorio Clínico, University Hospital Virgen de las Nieves, 18014 Granada, Spain; 6 Hematology Laboratory, Institut de Recerca Hospital Sant Joan de Déu, 08950 Barcelona, Spain; mcamos@hsjdbcn.org; 7 Centro de Investigación Biomédica en Red de Enfermedades Raras (CIBERER), ISCIII, 28029 Madrid, Spain; 8 Leukemia and Other Pediatric Hemopathies, Developmental Tumors Biology Group, Institut de Recerca Hospital Sant Joan de Déu, 08950 Barcelona, Spain; 9 Hematology Laboratory, Universitary Regional Hospital, Av. de Carlos Haya, 29010 Málaga, Spain; a.jimenez.velasco@gmail.com

**Keywords:** CTGF, CCN2, AL133346.1, lncRNA expression, biomarker, pediatric B-ALL

## Abstract

**Simple Summary:**

Dysregulation of noncoding RNAs has been described in numerous types of cancers and it has been associated with oncogenic or tumor suppressor activities. However, the signature of clinically relevant noncoding RNAs in pediatric B-cell acute lymphoblastic leukemia is still poorly understood. In a search for long non-coding RNAs that characterize pediatric B-cell acute lymphoblastic leukemia, we found that the long non-coding RNA AL133346.1 and a neighbouring protein-coding mRNA (CCN2) were significantly over-expressed in leukemia samples compared to healthy bone marrow. Survival analysis showed that patients with high CCN2 expression had a significantly better prognosis. These data suggest that AL133346.1/CCN2 could be useful for discriminating subtypes of leukemia and that CCN2 expression could predict the prognosis of pediatric patients with B-cell acute lymphoblastic leukemia.

**Abstract:**

Pediatric acute B-cell lymphoblastic leukemia (B-ALL) constitutes a heterogeneous and aggressive neoplasia in which new targeted therapies are required. Long non-coding RNAs have recently emerged as promising disease-specific biomarkers for the clinic. Here, we identified pediatric B-ALL-specific lncRNAs and associated mRNAs by comparing the transcriptomic signatures of tumoral and non-tumoral samples. We identified 48 lncRNAs that were differentially expressed between pediatric B-ALL and healthy bone marrow samples. The most relevant lncRNA/mRNA pair was AL133346.1/CCN2 (previously known as RP11-69I8.3/CTGF), whose expression was positively correlated and increased in B-ALL samples. Their differential expression pattern and their strong correlation were validated in external B-ALL datasets (Therapeutically Applicable Research to Generate Effective Treatments, Cancer Cell Line Encyclopedia). Survival curve analysis demonstrated that patients with “high” expression levels of CCN2 had higher overall survival than those with “low” levels (*p* = 0.042), and this gene might be an independent prognostic biomarker in pediatric B-ALL. These findings provide one of the first detailed descriptions of lncRNA expression profiles in pediatric B-ALL and indicate that these potential biomarkers could help in the classification of leukemia subtypes and that CCN2 expression could predict the survival outcome of pediatric B-cell acute lymphoblastic leukemia patients.

## 1. Introduction

Acute lymphoblastic leukemia (ALL) is the most common childhood malignancy. Pediatric B-cell ALL (B-ALL) constitutes a heterogeneous and aggressive neoplasia in which new targeted cancer therapies are required [1,2,3,4,5,6]. In recent years, novel genomic and transcriptomic techniques have accelerated the discovery and identification of various types of non-coding RNAs (ncRNAs) that may play an important role in cellular biology [7,8,9,10]. Long ncRNAs (lncRNAs) are transcripts longer than 200 bp that lack protein coding capacity. LncRNAs are crucial regulators of gene expression due to their involvement in post-transcriptional modifications of mRNAs, including splicing, editing, trafficking, translation, and degradation [11,12]. 

Dysregulation of lncRNAs has been described in numerous human diseases, including cancer [13,14,15,16,17], leading to oncogenic or tumor-suppressive activities, as we recently reviewed [18]. However, few dysregulated lncRNAs have been directly related to leukemia development. In acute myeloid leukemia (AML), chronic lymphocytic leukemia (CLL), and multiple myeloma (MM), HOTAIR was reported to be dysregulated, and its high expression was associated with reduced survival times in AML patients [19]. In t(8;21) positive AML, which is usually associated with a good prognosis, over-expression of CCAT1 and PVT1 was correlated with poor prognosis [20]. Even fewer studies have identified dysregulated expression of lncRNAs in pediatric B-ALL patients. TCL6 upregulation was recently associated with ETV6-RUNX1-positive pediatric B-ALL, and patients expressing low levels of TCL6 had lower disease-free survival than patients expressing high levels of TCL6 [21]. In another microarray-based study, the expression levels of BALR-2 were related to: (i) cytogenetic abnormalities such as t(12;21)[ETV6/RUNX1], t(1;19)[TCF3/PBX1] and MLL-rearranged; (ii) disease subtypes; and (iii) survival of B-ALL patients [22]. Interestingly, these studies and others have shown that lncRNA expression profiles can distinguish molecular leukemia subtypes [23,24,25,26,27,28].

Despite all the reported expression profiles, a consensus of a clinically relevant lncRNA signature for pediatric B-ALL is yet to be identified. Identifying a signature of differentially expressed lncRNAs and associated protein coding genes in pediatric B-ALL could lead to a better diagnosis, to a better understanding of the disease and, ultimately, to a better prognosis for the patients.

Previously, we developed a comparative study of the lncRNA profiles of pediatric B-ALL patients with and without the ETV6-RUNX1 gene fusion (Gene Expression Omnibus Accession GSE128254). We reported that high expression of the lncRNA TCL6 was associated with ETV6-RUNX1-positive pediatric B-ALL and with better disease-free survival, even within the ETV6-RUNX1-positive subtype [21]. Here, we performed a comparative study of the lncRNA profiles of pediatric B-ALL patients by leveraging data from our previous study as well as from multiple independent external cohorts.

## 2. Results and Discussion

### 2.1. Aberrantly Expressed lncRNAs in Pediatric B-ALL

**Figure 1:**
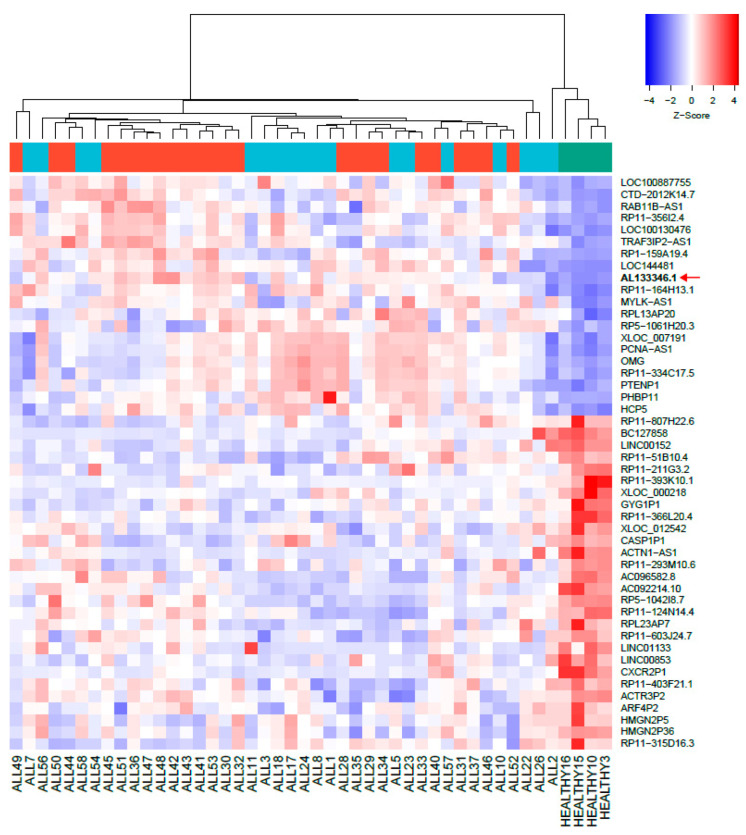
Heatmap of differentially expressed lncRNAs in B-ALL vs. healthy bone marrows. On top of the heatmap, ETV6-RUNX1-negative B-ALL samples are labeled in blue, ETV6-RUNX1-positive B-ALL samples are labeled in red, and healthy bone marrows are labeled in green. Clustering was performed based on the Spearman correlation coefficient.

**Figure 2:**
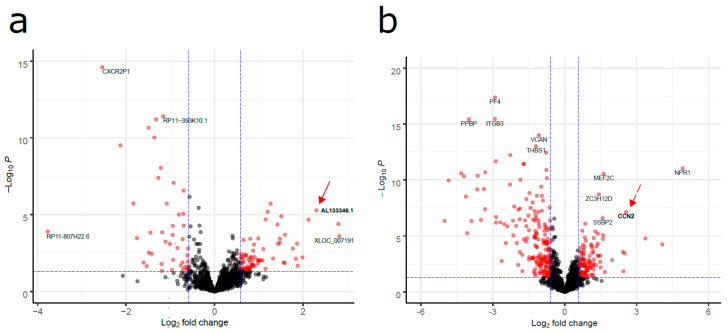
Volcano plot of the differentially expressed lncRNAs (**a**) and protein-coding genes (**b**) in pediatric B-ALL vs. healthy bone marrows. The horizontal dashed line represents a threshold of FDR = 0.05. The vertical dashed lines represent the thresholds of fold change = −1.5 and fold change = 1.5. Red dots represent the statistically significant differentially expressed lncRNAs.

To measure the lncRNA expression profiles in pediatric B-ALL, we compared lncRNA expression levels in 42 pediatric B-ALL patient samples against 4 healthy bone marrow samples (Figure 1). Despite the molecular heterogeneity of pediatric B-ALL, we identified a common differential expression profile that could distinguish the pediatric B-ALL samples from the healthy bone marrow samples. Setting a threshold of fold change > 1.5 and FDR < 0.05, we identified 48 lncRNAs that were differentially expressed between all B-ALL and healthy tissues, out of which 20 were upregulated and 28 were downregulated (Table S1). The top upregulated lncRNAs in pediatric B-ALL were XLOC_007191 (~7-fold), PCNA-AS1 (~7-fold) and AL133346.1 (~6-fold), whereas the top downregulated lncRNAs were RP11-807H22.6 (~14-fold), CXCR2P1 (~6-fold), and BC127858 (~4-fold) (Figure 2a). CCN2 was the 4th top upregulated protein-coding gene if we ranked by fold change and statistical significance (logFC = 2.56, FDR = 3.0 × 10^−6^) (Figure 2b).

**Figure A1:**
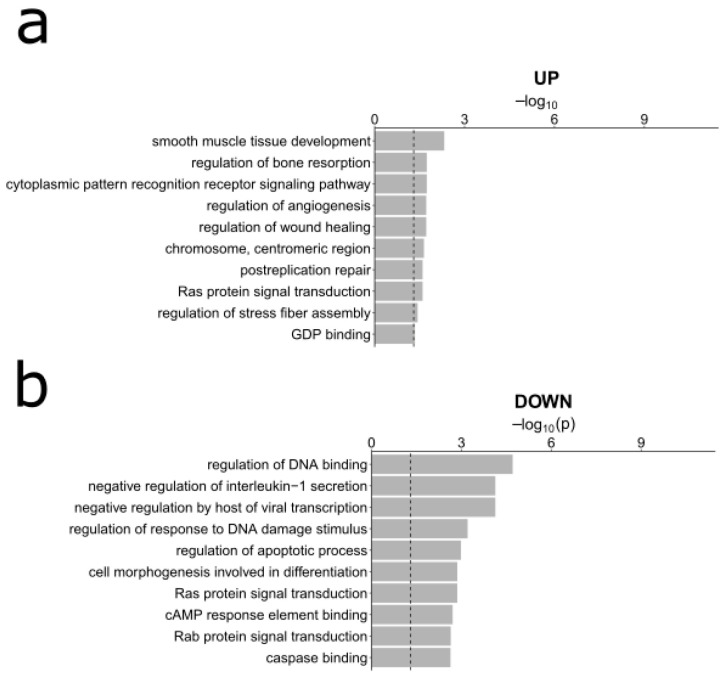
Gene Ontology (GO) analysis of the mRNAs associated with the differentially expressed lncRNAs between pediatric B-ALL and healthy samples. The top 10 significantly enriched upregulated (**a**) and downregulated (**b**) GO terms are shown. Only non-redundant GO terms are included as described in Methods.

We further investigated the putative biological functions of the differentially expressed lncRNAs between pediatric B-ALL and healthy bone marrow samples. Due to the lack of functional annotation of lncRNAs, we performed a Gene Ontology analysis on the protein-coding genes that were predicted to be functionally associated with the differentially expressed lncRNAs according to the microarray manufacturer. The top upregulated Gene Ontology terms included terms related to the regulation of angiogenesis (*p* = 0.019) (related genes: *ANGPTL4*, *NF1*, and *TNFAIP3*), DNA repair (*p* = 0.025) (*PCNA* and *UBE2V1*) and the RAS protein signal transduction pathway (*p* = 0.025) (*NF1*, *RAB4A*, and *RAB11B*) (Figure A1a). The top downregulated Gene Ontology terms included terms related to the negative regulation of interleukin-1 secretion (*p* = 7.36 × 10^−5^) (*CARD17* and *CARD18*), the regulation of response to DNA damage stimulus (*p* = 6.20 × 10^−4^) (*HMGA2* and *FEM1B*) and the regulation of apoptotic process (*p* = 1.04 × 10^−3^) (*ACTN1*, *EPHA1*, *HMGA2*, *JUN*, and *NOTCH2*) (Figure A1b). Taken together, these results suggest an overall oncogenic signature associated with lncRNA/mRNA expression profiles in pediatric B-ALL compared to healthy bone marrow samples. 

### 2.2. mRNA Levels of the AL133346.1/CCN2 Pair Are Increased in Pediatric B-ALL

Among the lncRNA/mRNA pairs that were differentially expressed between pediatric B-ALL and healthy bone marrows, AL133346.1/CCN2 (previously known as RP11-69I8.3/CTGF) were also upregulated in ETV6-RUNX1-positive vs. ETV6-RUNX1-negative pediatric B-ALL (fold change = 1.79, adjusted *p* = 0.036) [21]. In addition, both AL133346.1 and CCN2 were, individually, among the top differentially expressed lncRNAs and protein coding genes, respectively (Figure 2). In our previous analysis of multiple independent datasets, the expression levels of AL133346.1 and CCN2 were the most strongly correlated among all lncRNA/mRNA pairs [21]. Their differential expression patterns and their strong correlation led us to further investigate AL133346.1/CCN2 in additional external datasets.

**Figure 3:**
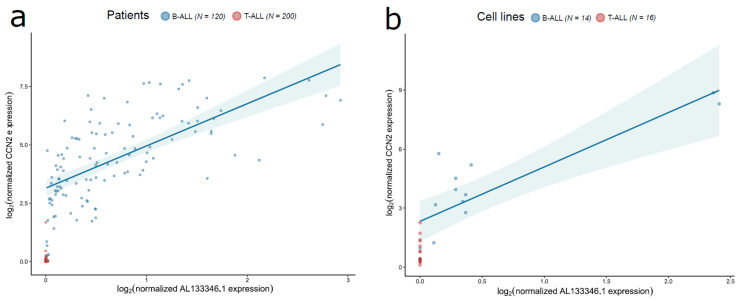
Comparison of AL133346.1/CCN2 expression between pediatric B-ALL and T-ALL samples from TARGET (**a**) and CCLE (**b**). The scatterplots are colored according to the ALL subtype. Sample sizes and gene expression correlation: TARGET 120 B-ALL (Spearman correlation coefficient = 0.717, *p* < 0.0001) vs. 200 T-ALL (Spearman correlation coefficient = 0.099, *p* = 0.163). CCLE: 14 B-ALL (Spearman correlation coefficient = 0.799, *p* = 0.001) vs. 16 T-ALL (Not available).

**Figure A2:**
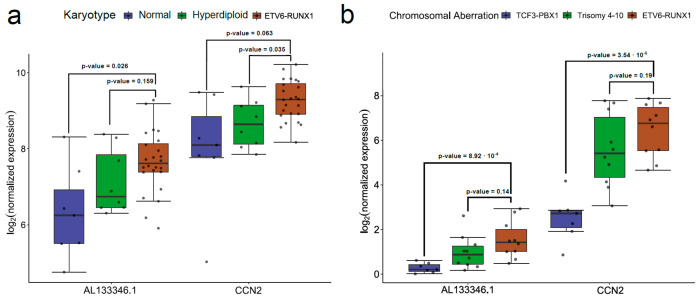
Comparison of AL133346.1 and CCN2 expression between different karyotypes of pediatric B-ALL patients from our own microarray (**a**) and different subtypes of pediatric B-ALL patients from TARGET (**b**)**.** The boxplots are colored according to the ALL subtype. The BCR-ABL and MLL subtypes had 3 and 0 positive samples respectively, thus they were excluded from the boxplot. Sample sizes: [TCF3-PBX1]: 7; Trisomy 4–10: 10; [ETV6-RUNX1]: 10.

We extended our analyses of AL133346.1/CCN2 to Therapeutically Applicable Research To Generate Effective Treatments (TARGET-ALL-P1 and TARGET-ALL-P2), which is, to our knowledge, the largest available collection of pediatric ALL gene expression data. After excluding patients who were older than 14.99 years and those without AL133346.1 and CCN2 expression data, the TARGET population consisted of 320 patients (120 B-ALL and 200 T-ALL). The B-ALL TARGET dataset mostly consisted of pre-B samples (112/120, 93.33%), whereas our cohort was more representative of B common phenotype (38/42, 90.48%). Because there were no better controls, we assessed the specificity of the expression of AL133346.1 and CCN2 by comparing their expression in B-ALL and in T-ALL. We found higher expression levels of AL133346.1 and CCN2 in B-ALL compared to T-ALL (Figure 3a). AL133346.1 and CCN2 were expressed in all analyzed subtypes of pediatric B-ALL, but they had the highest expression levels in ETV6/RUNX1-positive B-ALL compared to other subtypes of leukemia (Figure A2). We further confirmed our findings using ALL cell line data from the Cancer Cell Line Encyclopedia (CCLE) (Figure 3b). Overall, these results confirm that AL133346.1 and CCN2 are expressed in pediatric B-ALL and that their expression is low or absent in T-ALL.

### 2.3. AL133346.1/CCN2 Expression is Correlated in Pediatric B-ALL Samples

LncRNAs can regulate the expression of mRNAs by various mechanisms, which may result in a significant correlation pattern between the expression of the lncRNA and the associated mRNA [29]. We previously reported a strong positive correlation between the RNA levels of AL133346.1 and CCN2 in our cohort, as well as in external data from three different microarray studies in pediatric B-ALL patients [21]. To confirm our findings, we performed Spearman correlation analyses between the expression levels of AL133346.1 and CCN2 measured by RNA sequencing in B-ALL samples from TARGET and CCLE. We found a statistically significant correlation between AL133346.1 and CCN2 expression in TARGET pediatric B-ALL patients (*N* = 120, Spearman correlation coefficient = 0.717, *p* < 0.0001). In contrast, the expression levels of AL133346.1 and CCN2 were not correlated in pediatric T-ALL (*N* = 200, Spearman correlation coefficient = 0.099, *p* = 0.163), suggesting that the correlation is specific to B-ALL or that AL133346.1 is not expressed above the background in T-ALL. Similarly, we obtained a strong correlation in B-ALL cell lines from CCLE (*N* = 14, Spearman correlation coefficient = 0.799, *p* = 0.001), whereas this correlation was not found in T-ALL cell lines (*N* = 16) because AL133346.1 was not expressed in any T-ALL cell line. In addition, mRNA and protein levels of CCN2 are moderately correlated (Spearman correlation coefficient = 0.518, *p* = 0.001) according to data from Yang et al. [30].

To assess whether AL133346.1 could be involved in the regulation of other genes, we studied the pairwise correlations of AL133346.1 expression and expression of all genes (protein-coding and non-coding) in our microarray and in pediatric TARGET B-ALL data. In our own microarray, which included 1906 protein-coding genes, AL133346.1 expression was only significantly correlated with CCN2 expression. In TARGET B-ALL data, 9080 genes showed a statistically significant correlation with AL133346.1 expression in pediatric B-ALL data, and CCN2 was among the top 100. In any case, we note that correlation between these two genes, AL133346.1 and CCN2, does not necessarily mean that one of them modulates the other.

### 2.4. Analysis of Regulation Mechanisms of AL133346.1/CCN2

**Figure 4:**
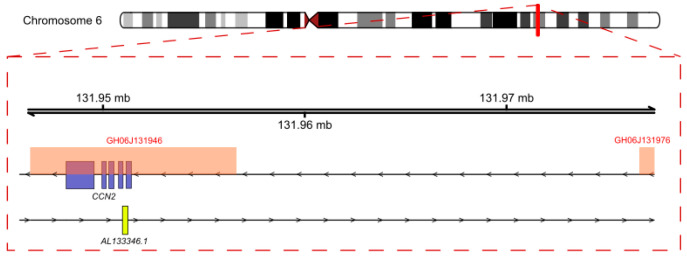
Genomic location of *AL133346.1*/*CCN2* in chromosome 6. The box with dashed stripes represents a zoom-in of the locus. All CCN2 exons are shown while only the first AL133346.1 exon is included. GH06J131946 and GH06J131976 regulatory regions are represented by red boxes.

Even if the expression of a lncRNA and its neighboring protein-coding gene are correlated, the lncRNA may not regulate its neighboring protein-coding gene. Landmark studies have shown that the regulatory function of certain lncRNA genes may not reside in the RNA product but in regulatory elements contained within the lncRNA gene, and that a common transcription factor may modulate the expression of both the lncRNA and the neighboring protein-coding gene resulting in correlated transcription [31,32]. To assess whether this phenomenon could explain the coexpression of AL133346.1 and CCN2, we studied the genomic location of the *AL133346.1* and *CCN2* genes and we searched for nearby promoters and proximal regulatory regions. *AL133346.1* and *CCN2* are located in the chromosome 6 (q23.2) but in different strands. The two genes are oriented head-to-head, partly overlapping, and their transcription start sites (TSS) are separated by 2770 pb. According to GeneHancer [33], which integrates promoter and enhancer data from various databases, *AL133346.1* and *CCN2* share two of their predicted high-score promoters/enhancers: GH06J131946 and GH06J131976 (Figure 4). These two enhancers are classified as high-likelihood enhancers (noted as “elite”) and they display a strong enhancer–gene association. The positive gene correlation results observed above in B-ALL point to the possibility that the protein-coding gene *CCN2* and its antisense lncRNA *AL133346.1* are expressed in a tissue-specific manner when their locus is transcriptionally active. Based on our results, we propose for our model that either: (i) AL133346.1 regulates CCN2 expression in cis; or (ii) *AL133346.1* and *CCN2* are specifically modulated in B-ALL by the same regulatory elements.

### 2.5. High Expression of CCN2 is Associated with Better Prognosis

**Figure A3:**
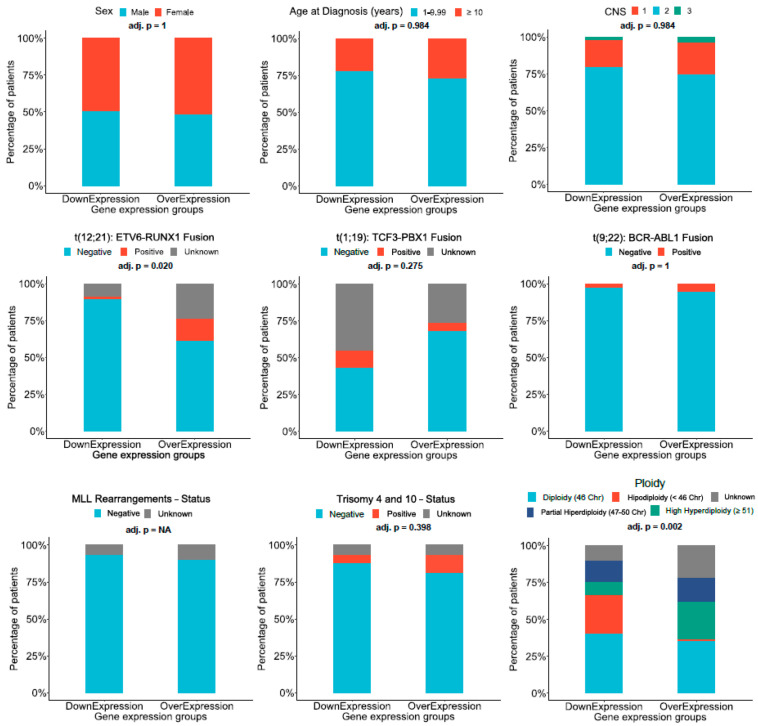
Comparison of clinical characteristics according to AL133346.1 expression in pediatric B-ALL patients from TARGET dataset. FDR-adjusted p-values of Fisher’s exact tests are shown. Abbreviations: Chr: Chromosome; CNS: central nervous system; CNS 1 means no CNS involvement, CNS 2 is a very low level, and CNS 3 is definite CNS involvement; NA: not available; MLL: *MLL* gene; t(12;21): translocation(12;21); t(1;19): translocation(1;19); t(9;22): translocation(9;22); (+): Positive; (−): Negative.

**Figure A4:**
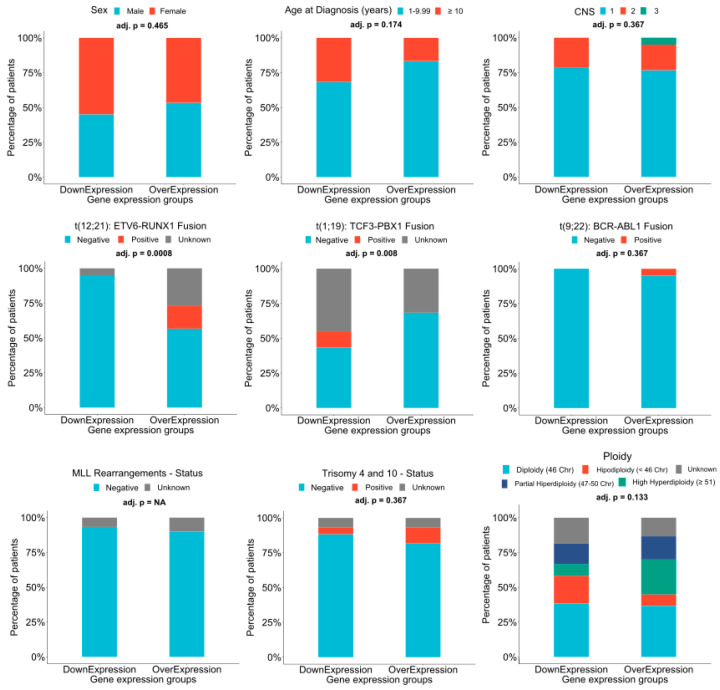
Comparison of clinical characteristics according to CCN2 expression in pediatric B-ALL patients from TARGET dataset. FDR-adjusted p-values of Fisher’s exact tests are shown. Abbreviations: Chr: Chromosome; CNS: central nervous system; CNS 1 means no CNS involvement, CNS 2 is a very low level, and CNS 3 is definite CNS involvement; NA: not available; MLL: *MLL* gene; t(12;21): translocation(12;21); t(1;19): translocation(1;19); t(9;22): translocation(9;22); (+): Positive; (−): Negative.

To determine the prognostic value of AL133346.1 and CCN2 expression, we performed a survival analysis using pediatric B-ALL clinical data from TARGET. The clinical information included gender, age at diagnosis, central nervous system involvement, t(12;21)[ETV6/RUNX1], t(1;19)[TCF3/PBX1], t(9;22)[BCR/ABL1], MLL rearrangement, trisomy of chromosomes 4 and 10 and number of chromosomes (Table S2). We found a statistically significant difference between “high” and “low” AL133346.1 and CCN2 expression subgroups and clinical covariates (Figure A3, Figure A4, Table S3). Two of the most reliable clinical parameters to determine aggressiveness, the t(12;21)[ETV6/RUNX1] and the t(1;19)[TCF3/PBX1] translocations [34], were not distributed homogeneously in the analysed subgroups. We observed that the “high” AL133346.1 and CCN2 expression subgroups included 90% and 100% of the t(12;21)[ETV6-RUNX1]-positive B-ALL patients, whereas they had 29% and 0% of the t(1;19)[TCF3-PBX1]-positive cases, respectively (Figure A3, Figure A4, Table S3).

**Figure 5:**
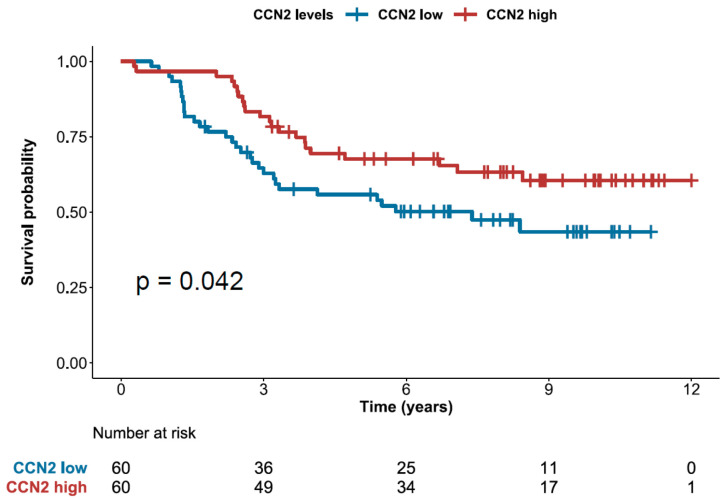
Kaplan-Meier overall survival curves of pediatric B-ALL patients divided in two groups according to whether CCN2 expression was above or beyond the median: “CCN2 high” and “CCN2 low”. The *p*-value of Cox univariate analyses is shown.

**Figure A5:**
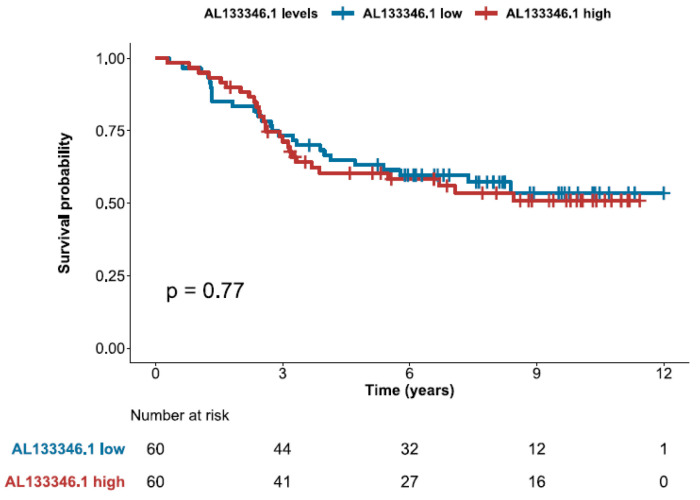
Kaplan-Meier overall survival curves of pediatric B-ALL patients divided in two groups according to whether AL133346.1 expression was above or beyond the median: “AL133346.1 high” and “AL133346.1 low”. The p-value of Cox univariate analyses are shown.

The univariate Cox analyses for variables traditionally related with survival in pediatric B-ALL revealed that male gender (*p* = 0.030), TCF3-PBX1 gene fusion (*p* < 0.001) and ploidy (Hipodiploidy: *p* = 0.005; Partial Hiperdiploidy: *p* = 0.033; High Hiperdiploidy: *p* = 0.019) were significantly associated with a shorter survival, while age at diagnosis (*p* = 0.186), CNS involvement (*p* = 0.943), ETV6-RUNX1 gene fusion (*p* = 0.490), BCR-ABL1 gene fusion (*p* = 0.689) and trisomy of chromosomes 4 and 10 (*p* = 0.418) were not statistically significant in this cohort. Splitting the patients by their median AL133346.1 or CCN2 expression, we found that “high” CCN2 patients were associated with longer overall survival (OS) times (*p* = 0.042; HR: 0.566, 95% CI: 0.328–0.980) (Figure 5) while there was no association between OS and AL133346.1 expression (*p* = 0.77) (Figure A5). Although AL133346.1 expression and CCN2 expression were significantly correlated, and CCN2 was the top protein coding gene correlated with AL133346.1 in our microarray, only CCN2 was significantly associated with OS. This fact can be explained because the correlation between AL133346.1 and CCN2 is not perfect. This suggests that AL133346.1 may participate in CCN2-independent biological processes, which would interfere with the association of AL133346.1 with OS. In addition, all clinical covariates with *p* < 0.2 in a Cox univariate analysis, together with CCN2 expression, were used for a Cox multivariate analysis. The TCF3-PBX1 gene fusion was the most important variable influencing OS in pediatric B-ALL (*p* = 0.003). These results revealed that “high” CCN2 expression could be a prognostic factor associated to longer OS (*p* = 0.045) (HR: 0.448, 95%CI: 0.204–0.984) (Table S4). Although the “high” CCN2 expression subgroup was significantly enriched in t(12;21)[ETV6-RUNX1]-positive (*p* = 0.0001) and t(1;19)[TCF3-PBX1]-negative (*p* = 0.002) B-ALL patients (Table S3), we found no association between t(12;21)[ETV6/RUNX1] and OS, and we accounted for t(1;19)[TCF3-PBX1] in the Cox multivariate model. Thus, the expression of CCN2 could distinguish between two clinically different subgroups independently of the translocations in the sample.

**Figure A6:**
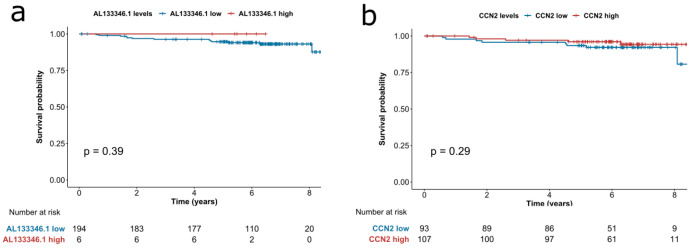
Kaplan-Meier overall survival curves of pediatric T-ALL patients divided in two groups according to whether AL133346.1 (**a**) and CCN2 (**b**) expression was non-null or null: “AL133346.1 high”, “AL133346.1 low”, “CCN2 high” and “CCN2 low”. The p-values of Cox univariate analysis are shown.

Finally, to assess whether CCN2 could be a specific prognostic biomarker of pediatric B-ALL, we performed a survival analysis using T-ALL clinical data (Table S5). “High” CCN2 expression was not associated (*p* = 0.29) with a survival benefit for patients with T-ALL (Figure A6a), indicating a specific role of CCN2 in pediatric B-ALL. Moreover, there was no association between OS and AL133346.1 expression (*p* = 0.39) in pediatric T-ALL patients (Figure A6b).

CCN2 belongs to the Cellular Communication Network (CCN) family of proteins. This family of extracellular matrix-associated proteins is characterized by having four conserved cysteine-rich domains and is involved in intercellular signaling [35]. In hematopoietic malignancies the CCN family plays a significant role in the differentiation of hematopoietic stem cells (HSC) and mesenchymal stem cells [36]. The prognosis value of the CCN family of proteins varies in the different hematologic malignancies [36]. In agreement with our results, several studies significantly correlated the expression of CCN2 with greater overall survival in lymphoma treated with chemotherapy [37,38]. However, further specific studies are needed in pediatric B-ALL to determine if CCN2 expression levels could influence patient treatment outcome.

## 3. Materials and Methods

Unless specified otherwise, all analyses were performed using R (version 4.0.1) and Bioconductor (version 3.11).

### 3.1. Statistical Analyses

Normality of continuous data was assessed using quantile-quantile plots and the Shapiro-Wilk test. For normal data, mean and standard deviation were reported and two-tailed Student’s *t*-tests were applied after checking for equality of variances. For non-normal data, the median and quartiles were reported and two-sided Wilcoxon rank-sum tests were applied. Whenever applicable, multiple comparisons adjustments were performed using the Benjamini-Hochberg method.

### 3.2. Microarray Data Analyses

Raw and normalized gene expression values for each sample in the analysis from our previous microarray study [21] are publicly available at the Gene Expression Omnibus (GSE128254). The methods for sample processing, microarray hybridization and data analysis are fully detailed in the section “Data pre-processing and differential expression analyses” Supplementary Methods of the original publication [21]. The differentially expressed lncRNAs/mRNAs between tumor and normal samples were obtained by false discovery rate (FDR) < 0.05 and fold change (absolute value) > 1.5. Then, we performed a hierarchical clustering based on the Spearman correlation coefficient to identify lncRNA and mRNA expression patterns among tumoral and healthy bone marrow samples.

### 3.3. Gene Ontology Analyses

We performed Gene Ontology analyses to identify significantly enriched biological pathways, molecular functions or cellular components related to the differentially expressed lncRNAs identified in the microarray analyses between tumor and normal samples (FDR < 0.05, fold change (absolute value) > 1.5). The protein-coding genes associated to the differentially expressed lncRNA/mRNA pairs were predicted by the microarray manufacturer based on their genomic proximity, predicted miRNA binding, and the scientific literature as described in “Microarray design and predicted regulation between lncRNAs and protein-coding genes” section of the Supplementary Methods of our previous manuscript [21]. Using these predictions, we performed the Gene Ontology analysis as follows:

- Find the significant upregulated or downregulated lncRNAs in our analysis of pediatric B-ALL vs. healthy bone marrow. We analyzed up- and downregulated lncRNAs separately.
- Using the lncRNA/mRNA predictions, find the mRNAs that were predicted to be associated with the selected lncRNAs.
- Enter the mRNA list as input to Enrichr [39,40] to find significantly enriched Gene Ontology terms.
- Simplify the Gene Ontology terms using ReviGO [41] with default parameters and providing the FDR values for each GO term.

### 3.4. Analysis of External Datasets

We downloaded gene expression of ALL patients from Genomic Data Commons (TARGET Project, last updated 6 June 2020; *N* = 506). Data were downloaded using the R package TCGABiolinks (v2.16.0). In the case of samples from the same patient, those with the smallest library size were removed (*N* = 477). For the expression data, raw counts per gene were normalized using the TMM method and then log2(counts per million) were calculated using the R package edgeR (v3.30.3). For the survival analysis, patients who were more than 14.99 years old were filtered out (*N* = 321). Then, B-subtype (*N* = 120) and T-subtype (*N* = 200) samples were used to perform the following analysis. In this case, the B-subtype dataset consisted of pre-B (112/120, 93.33%) and B-ALL (8/120, 6,67%) samples, while all T-subtype samples were T-ALL.

In addition, we downloaded external gene expression of ALL cell lines from Cancer Cell Line Encyclopedia (https://portals.broadinstitute.org/ccle/data). TPM-normalized gene expression data were directly downloaded from the CCLE data repository (version 2019-09-29, *N* = 1019). Using cell line sample information from DepMap portal, we selected B-ALL (*N* = 14) and T-ALL (*N* = 16) cell lines. Normalized gene expression data were transformed to log2 scale for the following analyses. Then these transformed data were filtered to obtain AL133346.1 and CCN2 expression.

The downloaded data from both databases were used for comparison of gene expression between both subtypes and to study the lncRNA/mRNA correlation using the Spearman correlation coefficient.

### 3.5. Survival Analyses

The clinical data associated with TARGET ALL patients were downloaded from NCI TARGET (Therapeutically Applicable Research To Generate Effective Treatments) repository (https://target-data.nci.nih.gov/Public/ALL/clinical/). We used AL133346.1 and CCN2 expression levels from TARGET data to analyze their correlation with overall survival of ALL patients, considering deaths as events. We split the patients in two groups for both genes according to their expression levels. These gene expression variables were dichotomized into “low” and “high” subgroups using the median expression value for CCN2 and AL133346.1 as a threshold for B-ALL. These variables were dichotomized into the same levels if the expression values were null or non-null for T-subtype. The association between clinical characteristics and gene expression groups was determined using Fisher’s exact tests. We plotted Kaplan-Meier curves for “High” and “Low” patients groups for both genes using the R packages “survival” (v3.1-11) and “survminer” (v0.4.6), and significance was tested using the univariate log-rank test. In addition, we studied the effect of different clinical covariates on the overall survival patients performing univariate Cox regression. Those clinical covariates that showed *p* < 0.2 in univariate analysis were used for a multivariate Cox regression.

## 4. Conclusions

In conclusion, we observed increased AL133346.1 and CCN2 expression in pediatric B-ALL. Importantly, the stratification based on CCN2 expression could be of clinical interest, since our results revealed that “high” CCN2 expression was associated with better OS of pediatric B-ALL patients. We suggest that these potential biomarkers would further help in the classification of leukemia subtypes and the determination of the clinical outcome of pediatric B-ALL patients.

## Supplementary Materials

The following are available online at https://www.mdpi.com/2072-6694/12/12/3803/s1, Table S1: 48 lncRNAs, represented by 50 probes, that were differentially expressed between pediatric B-ALL vs. healthy bone marrow samples; Table S2: Demographic and clinical characteristics of the pediatric B-ALL patients of the TARGET dataset; Table S3: Comparison of clinical characteristics according to AL133346.1 and CCN2 expression in pediatric B-ALL patients from TARGET dataset; Table S4: Cox Univariate and multivariate overall analysis of clinical covariates in pediatric B-ALL patients from TARGET dataset; Table S5: Demographic and clinical characteristics of the pediatric T-ALL patients of the TARGET dataset.
