# Supplementary material for: LncRNA-mRNA Co-Expression Analysis Identifies AL133346.1/CCN2 as Biomarkers in Pediatric B-Cell Acute Lymphoblastic Leukemia
Source: Cancers (Basel). 2020 Dec 17;12(12):3803. doi: 10.3390/cancers12123803 (PMC7765782; doi:10.3390/cancers12123803)
Supplement: Supplementary file 1 [file cancers-12-03803-s001.pdf]

cancers

Supplementary materials: Lncrna-mrna Co-Expression Analysis Identifies AL133346.1/CCN2 as Biomarkers in Pediatric B-Cell Acute Lymphoblastic Leukemia

Marta Cuadros, Daniel J. García, Alvaro Andrades, Alberto M. Arenas, Isabel F. Coira, Carlos Baliñas-Gavira, Paola Peinado, María I. Rodríguez, Juan Carlos Álvarez-Pérez, Francisco Ruiz-Cabello, Mireia Camós, Antonio Jiménez-Velasco and Pedro P. Medina

Table S1. 48 lncRNAs, represented by 50 probes, that were differentially expressed between pediatric B-ALL vs. healthy bone marrow samples[4]. Overexpressed lncRNAs are in red and downregulated lncRNAs are in green. The lncRNAs are ranked by fold change (FC). FDR: false discovery rate.

GeneName	FC	*P*-Value	FDR	seqname	source	Probe sequence
XLOC_007191	7.0108201	0.000330928	0.012455896	TCONS_00015148	Cabili et al	AGCTGCCTGCTTAGAAGGACTTTGGAAGCAAGACAGAGCAGTGTCT
PCNA-AS1	6.8685316	6.63E-05	0.003170658	NR_028370	RefSeq	GAAAGACAACGACCACTCTGCTACGCCTGCAACCGTTTAATGCCGC
AL133346.1	5.1548851	2.32E-05	0.001477978	ENST00000435287	gencode	GTAACTTGGACAACCTGAGGCATTAATTGAGGATCAATATGATGAC
HCP5	4.4955343	4.06E-05	0.002302832	NR_040662	RefSeq	GCCCTGAGGCAAACTATGAATTAAAGTGTTGTGGATCCCACATGAA
LOC144481	4.1514389	4.28E-06	0.000436973	uc001tcu.3	UCSC_knowngene	TATTTTACCACAGGTACCAATCACCCTCCAGATCTAAGAATGGCTT
RP11-334C17.5	4.0109666	0.000581137	0.017794409	ENST00000573346	gencode	GGACCACTGCCTGCACGCCTTGGTCAAAACAAAGCCACACACCAGA
OMG	3.6061205	0.00101954	0.028380288	ENST00000584094	gencode	CAACTGCCTGCATTCTTATCTCAGTTTCTTATTATTGCTGAATGAC
RP1-159A19.4	3.1364878	0.000534767	0.017275697	ENST00000443579	gencode	GGTTTGAATGTACTCACTGAGTGGTCTTCATAAATTACCTCTCTGC
RP11-164H13.1	3.0092288	8.38E-06	0.000725088	ENST00000553445	gencode	TTCAGCCTGTGACTGCCCACCAGAGATGTCTTCCTGCGGCTAGCTG
LOC100130476	2.8915131	1.81E-05	0.001206789	NR_049793	RefSeq	CTGATGTAACTGCTTTGTCATCTTGCCTGCCAGGATTTGTGACAAG
RP11-356I2.4	2.8562064	0.000568603	0.017765957	ENST00000448942	gencode	CTCACTTCATAAGAGCAGTAAACACTAAACCAAGGGAATGTGAATT
LOC100887755	2.7318975	0.0008791	0.025394391	NR_051976	RefSeq	GGATGCCTGCTACTGTCACTGTTGTCATTAGATAATTAATGAACTA
PTENP1	2.6858211	5.15E-05	0.002627005	NR_023917	RefSeq	TGTGAATGCTTCATGTGCTGCCTGCAAGCTTCTTTTTTCTCATTAA
TRAF3IP2-AS1	2.6169707	0.000458242	0.015590427	NR_034110	RefSeq	TCAAGAAGATCAGAACAGGCTTAAATTGATGCTATAAAACCAATTA
CTD-2012K14.7	2.3007983	0.0001698	0.007427541	ENST00000562846	gencode	GACCGTTCCGTTGTGAGAATGCTGGCCCAATAACATACTCTTTTTT
MYLK-AS1	2.0228068	8.91E-05	0.004135494	NR_038266	RefSeq	GAATGACTTTGTGACCAGCAGCTTAAGCCAAATAAACTTTATAAGG
PHBP11	1.9510706	0.000941069	0.02668106	ENST00000420550	gencode	ACAGAAACCAATTATCTTTGACTGCCGTTCTCGACCACGTAAAGTG
RP11-164H13.1	1.9465779	1.04E-05	0.000773313	ENST00000547644	gencode	ACCATACATCAGTCATCTGGCAGCAGCTCGAGAAGCATGTCCTCCA
RAB11B-AS1	1.895501	0.001389946	0.036067912	NR_038237	RefSeq	TCACTTTTTAAAACAAACTGAGGGTAACTTACTAGATCTGTGAACT
RP5-1061H20.3	1.7178744	0.001668236	0.040540785	ENST00000434404	gencode	AGAGCGGCAGGTGGGGGCGTTGGGGGCTGAGTCCCGATTTCCCTGA
RPL13AP20	1.5736643	0.000422041	0.015089406	NR_003932	RefSeq	AGAAGAAAATTGACAAATACACAGAAGTCCTCAAGACCCACGGACT
RP11-807H22.6	-13.874405	0.000150882	0.006794133	ENST00000539482	gencode	CCCTGGGGTTCTTCCGACAACCTATCCTAATAGACAAATCCACATG
CXCR2P1	-5.7957804	0	0	NR_002712	RefSeq	GTACTTTTGCCAAAGAACCAAGGCTGAAATTTCTTGCTAAATGAGA
BC127858	-4.4412668	1.19E-09	0.00000026	uc002zti.1	UCSC_knowngene	AAACACCCAAGTGTTAGGAACCAGAATAATCAGCCCTAATAGAGGG

LINC00152	-3.90121	0.000000062	0.000010547	NR_024206	RefSeq	TTGAATAACTGGGAGATGAAACAGGAAGCTCTATGACACACTTGAT
ACTN1-AS1	-3.6890357	7.00E-11	0.000000027	ENST00000553944	gencode	TCTAAACTGTGGCTCTAAACCTAACACAGAGGGTAATGGTAGTGCT
RP11-124N14.4	-3.520628	3.96E-06	0.000433239	ENST00000437232	gencode	TGTTTCGAAGTCGATGCAAAAAATGAGTTGCTTATTTCAGTCTCTC
RP11-51B10.4	-3.3449726	0.000444639	0.015471426	ENST00000451920	gencode	TGCAGGGACCTCTTTATCATCCATGGCAAGGATGTCCTTCCCCAGA
AC092214.10	-2.7754398	1.30E-10	0.00000004	ENST00000421648	gencode	AGTGTGTATTCAGATACAAGCCTTTATCTCAATACCTTCCTTACCC
ACTR3P2	-2.707763	0.000213907	0.008851145	ENST00000439585	gencode	TGAGTGGCAGTAGATTGAAGCCAAAATCTATTGATGTACAAGTCGT
RP11-366L20.4	-2.5473442	2.20E-10	0.000000056	ENST00000536217	gencode	GGTTTCCTGTTCCTTTTGCTTCCTGGAAAATACAGACCTACAAAGC
XLOC_000218	-2.4826843	1.00E-11	0.000000005	TCONS_00000959	Cabili et al	CATCTTAGTGTGACTAAAAGAGGGAGATCCTAAAGAATTTCAAGGA
GYG1P1	-2.4041661	4.72E-08	0.000009027	ENST00000460864	gencode	CAAAAAGTGTCAAAAGTGATTCCCATGATCCCAACATAACTTATCC
HMGN2P36	-2.3071326	0.000199267	0.008474388	ENST00000529541	gencode	GAGATGCCAAGTGAAGTATGTGCATTTTTGATAACTGTGCACTCCT
RP11-393K10.1	-2.2373338	0	0	ENST00000431627	gencode	GCACAATATCAAGTGTGAGGAAGGAGATAAGTTTGATTATTCTGC
HMGN2P5	-2.2154239	0.001250201	0.033579954	ENST00000436882	gencode	CCAAAACAGACCAGGCACAGAAAGCTGAAGGTGCTGGAGATGCCAA
RP5-1042I8.7	-2.0775564	1.40E-05	0.000976553	ENST00000566949	gencode	TAGTGTGTTTCTTCACCACTTTGGCTTTGAGCACTCAAAGTGCAGA
RP11-603J24.7	-2.0246615	0.000871451	0.025394391	ENST00000552016	gencode	GTACTGACCTGAAAACTTGTGACATAAAGAACACCAACAAGTGCTC
RP11-293M10.6	-1.9464185	0.000252757	0.010183432	ENST00000560419	gencode	GGCTTCTCTTCCAATGACCACCAATGAGAATGTTATAAAATATGTC
LINC01133	-1.9330405	5.52E-05	0.002727191	NR_038849	RefSeq	GCTTTGTATGTTATTAAATGTGTCTCGTCAATGCTGTTGGCATTGT
XLOC_012542	-1.9200927	1.86E-06	0.00021877	TCONS_00025724	Cabili et al	GCGGGAGCTGGTGCCCTGGCAGGAGGGACTGGCTTATGCCACCCTG
RP11-315D16.3	-1.8115402	0.001743703	0.041712656	ENST00000566875	gencode	ATGAAGGACAAACCACAGAGAAGATCCACGAGGTTTTCTGCTAAAC
CASP1P1	-1.8063674	0.000541629	0.017275697	ENST00000526345	gencode	TGAAAGGATGACTTTGACAATACGCTTCTACCTCTTCCCAGAACAT
RPL23AP7	-1.7817485	1.06E-05	0.000773313	NR_024530	RefSeq	TTCCACACTACAGAAAGAATCCCAGTCTTCTTAGAGAACCCAGTGA
RP11-403F21.1	-1.6433331	0.000423804	0.015089406	ENST00000443069	gencode	TTCTACATAAACCGAGTCAGCCACCAGATTCAGACCTTTGTGACAA
RP11-211G3.2	-1.6278029	4.41E-05	0.002330142	ENST00000450760	gencode	CACATAGAAAACTTGGAGCCAAAGCATTTGGCAAGAGCGGAAAAAA
LINC00853	-1.6215043	1.76E-07	0.000026917	NR_047498	RefSeq	GGATCCTTGAAGCACAATAAACAGATAGTATTTCTGCATGTGTCAA
AC096582.8	-1.621083	1.05E-05	0.000773313	ENST00000413839	gencode	TCTGAGCCAGCTGCACGACAAGTTCGAGCATCTTAAAATGATTCAA
RPL23AP7	-1.6020753	4.34E-05	0.002330142	NR_024531	RefSeq	AGACATTTAGGAGAAGAATCCCAGTCTTCTTAGAGAACCCAGTGAC
ARF4P2	-1.5143237	0.001450419	0.03640315	ENST00000430209	gencode	TCTTCAGTCTCTTCCTAACAGGACATGGTATGTTCAAGCCACTTGT

Table S2. Demographic and clinical characteristics of the pediatric B-ALL patients of the TARGET dataset. Abbreviations: Abbreviations: CNS: central nervous system; CNS 1 means no CNS involvement, CNS 2 is a very low level, and CNS 3 is definite CNS involvement. F: female; M: male; NA: not available; MLL: MLL gene; OS: Overall Survival; t(12;21): translocation(12;21); t(1;19): translocation(1;19); t(9;22): translocation(9;22); (+): Positive; (-): Negative.

Sample	Sex	Age at Diagnosis (years)	Phenotype	CNS	t(12;21): ETV6-RUNX1 Fusion	t(1;19): TCF3-PBX1 Fusion	t(9;22): BCR-ABL1 Fusion	MLL Rearrangements - Status	Trisomy 4 and 10 - Status	№ Chromosomes	Vital Status	OS Time (years)
TARGET-10-PAMDRM	M	7.87	B Cell ALL	2	(-)	(-)	(-)	(-)	(-)	46	Dead	3.32
TARGET-10-PAMXHJ	M	6.08	B-Precursor	2	(-)	(-)	(-)	(-)	(+)	60	Alive	11.20
TARGET-10-PAMXSP	M	3.35	B-Precursor	3	(-)	(-)	(-)	(-)	(-)	54	Alive	12.00
TARGET-10-PANATY	F	4.22	B-Precursor	1	NA	(-)	(-)	(-)	(-)	45	Alive	11.16
TARGET-10-PANDBX	F	3.83	B-Precursor	1	(-)	(-)	(-)	(-)	(-)	46	Dead	4.71
TARGET-10-PANEUH	M	1.77	B-Precursor	1	NA	(-)	(-)	(-)	(+)	55	Alive	11.31
TARGET-10-PANJPG	F	13.17	B-Precursor	1	NA	(-)	(-)	(-)	(-)	46	Alive	11.15
TARGET-10-PANJWJ	M	11.00	B-Precursor	3	NA	(-)	(+)	(-)	(-)	46	Alive	10.09
TARGET-10-PANKAK	M	6.41	B-Precursor	2	(-)	(-)	(-)	(-)	(+)	67	Dead	3.00
TARGET-10-PANKGK	M	12.74	B-Precursor	1	NA	(-)	(-)	(-)	(-)	46	Alive	5.57
TARGET-10-PANKMB	F	8.21	B-Precursor	2	NA	(-)	(-)	(-)	(-)	47	Alive	11.43
TARGET-10-PANLIC	F	14.84	B-Precursor	2	(-)	(-)	(-)	(-)	(-)	47	Alive	10.99
TARGET-10-PANNGL	F	12.85	B-Precursor	1	(-)	(-)	(-)	(-)	(-)	47	Alive	6.88
TARGET-10-PANRWG	M	2.68	B-Precursor	1	(-)	(-)	(-)	(-)	(-)	47	Alive	10.32
TARGET-10-PANRYM	F	2.33	B-Precursor	1	NA	(-)	(-)	(-)	(+)	55	Alive	4.59
TARGET-10-PANSDA	F	12.64	B-Precursor	1	NA	(-)	(-)	(-)	(-)	46	Dead	3.12
TARGET-10-PANSFD	M	5.44	B Cell ALL	2	(-)	(-)	(-)	(-)	(-)	46	Dead	1.54
TARGET-10-PANSHK	M	2.29	B Cell ALL	1	(-)	(-)	(-)	(-)	(-)	46	Dead	6.70
TARGET-10-PANSIA	F	5.59	B-Precursor	1	NA	(-)	(-)	(-)	(+)	64	Alive	10.76
TARGET-10-PANSPW	M	13.14	B-Precursor	1	NA	(-)	(+)	(-)	(-)	46	Dead	2.38
TARGET-10-PANSUL	M	2.43	B-Precursor	1	NA	(-)	(-)	(-)	(-)	46	Dead	2.92
TARGET-10-PANSXG	F	7.60	B-Precursor	2	(-)	(-)	(-)	(-)	(-)	53	Alive	10.49

TARGET-10-PANTSM	M	7.09	B-Precursor	1	(-)	(-)	(-)	(-)	(-)	45	Dead	3.32
TARGET-10-PANTTZ	F	2.60	B-Precursor	1	(-)	NA	(-)	(-)	(-)	48	Alive	9.97
TARGET-10-PANUHA	M	3.56	B-Precursor	1	(+)	(-)	(-)	(-)	(-)	46	Alive	11.00
TARGET-10-PANUUF	F	11.03	B-Precursor	1	NA	(-)	(-)	(-)	(-)	46	Alive	10.71
TARGET-10-PANWEZ	F	14.34	B-Precursor	1	(+)	(-)	(-)	(-)	(-)	46	Alive	8.61
TARGET-10-PANWHJ	M	3.10	B-Precursor	1	(-)	(-)	(-)	(-)	(-)	53	Alive	10.32
TARGET-10-PANWHW	F	4.02	B-Precursor	2	(-)	(-)	(-)	(-)	(-)	46	Alive	5.25
TARGET-10-PANWVW	M	2.95	B-Precursor	1	NA	(-)	(+)	(-)	(-)	47	Alive	9.77
TARGET-10-PANWYH	M	4.89	B Cell ALL	1	(-)	(-)	(-)	(-)	(-)	46	Dead	3.88
TARGET-10-PANXDR	M	3.26	B-Precursor	1	NA	(-)	(-)	(-)	(-)	57	Alive	10.41
TARGET-10-PANYEJ	M	1.86	B-Precursor	1	NA	NA	(-)	NA	NA	NA	Alive	10.61
TARGET-10-PANYGB	F	7.63	B Cell ALL	1	(-)	(-)	(-)	(-)	(-)	46	Dead	5.78
TARGET-10-PANYZE	F	4.05	B Cell ALL	1	(-)	(-)	(-)	(-)	(-)	50	Dead	8.39
TARGET-10-PANZPJ	M	13.02	B-Precursor	1	NA	NA	(-)	NA	NA	NA	Dead	2.61
TARGET-10-PAPAGK	F	2.34	B-Precursor	1	(-)	NA	(-)	NA	NA	NA	Alive	9.66
TARGET-10-PAPAIZ	F	6.45	B-Precursor	1	(-)	(-)	(-)	(-)	(-)	52	Alive	9.94
TARGET-10-PAPAXK	M	9.56	B-Precursor	1	(-)	NA	(-)	(-)	(-)	44	Alive	10.38
TARGET-10-PAPBCI	M	9.92	B-Precursor	2	NA	(-)	(-)	(-)	(-)	46	Dead	8.45
TARGET-10-PAPBFN	M	10.89	B-Precursor	1	NA	(-)	(-)	(-)	(+)	52	Dead	4.13
TARGET-10-PAPBSY	M	2.42	B-Precursor	1	(-)	(+)	(-)	(-)	(-)	46	Dead	1.82
TARGET-10-PAPCUR	F	3.01	B-Precursor	1	(-)	(-)	(-)	(-)	(-)	46	Dead	1.01
TARGET-10-PAPDKJ	M	3.97	B-Precursor	1	(+)	(-)	(-)	(-)	(-)	46	Dead	3.99
TARGET-10-PAPDUF	M	3.33	B-Precursor	1	(-)	(-)	(-)	(-)	(+)	56	Alive	10.05
TARGET-10-PAPDUV	F	5.44	B Cell ALL	1	(-)	(-)	(-)	(-)	(-)	46	Alive	9.59
TARGET-10-PAPECF	F	2.15	B-Precursor	1	(+)	(-)	(-)	(-)	(-)	48	Alive	3.53
TARGET-10-PAPEFH	F	6.96	B-Precursor	1	(-)	(-)	(-)	(-)	(-)	48	Dead	2.33
TARGET-10-PAPEJN	M	3.09	B Cell ALL	1	(+)	NA	(-)	NA	NA	NA	Dead	2.56

TARGET-10-PAPESW	F	2.89	B-Precursor	1	(-)	(-)	(-)	(-)	(-)	57	Alive	9.70
TARGET-10-PAPEWB	F	7.13	B-Precursor	2	(-)	(-)	(-)	(-)	(-)	46	Alive	9.39
TARGET-10-PAPFXN	F	1.84	B-Precursor	3	(-)	(-)	(-)	(-)	(-)	55	Alive	3.18
TARGET-10-PAPGFP	F	9.63	B-Precursor	1	(-)	(-)	(-)	(-)	(-)	46	Dead	2.59
TARGET-10-PAPGYC	F	3.66	B-Precursor	1	(-)	(-)	(-)	(-)	(-)	48	Dead	5.48
TARGET-10-PAPHGD	M	1.78	B-Precursor	1	(-)	(-)	(-)	(-)	(-)	45	Alive	9.80
TARGET-10-PAPHJF	F	6.87	B-Precursor	1	(-)	(+)	(-)	(-)	(-)	46	Dead	1.32
TARGET-10-PAPHYN	M	5.69	B-Precursor	2	(-)	(-)	(-)	(-)	(-)	46	Alive	8.25
TARGET-10-PAPHZT	F	1.51	B-Precursor	1	NA	(-)	(-)	(-)	(-)	46	Alive	9.29
TARGET-10-PAPIGX	F	14.32	B-Precursor	1	(-)	NA	(-)	NA	NA	NA	Alive	1.76
TARGET-10-PAPIJM	M	10.89	B-Precursor	1	(-)	NA	(-)	NA	(+)	NA	Alive	3.29
TARGET-10-PAPIRZ	F	13.39	B-Precursor	1	(-)	NA	(-)	NA	NA	NA	Dead	2.76
TARGET-10-PAPJHB	F	5.49	B-Precursor	1	(-)	(-)	(-)	(-)	(-)	47	Alive	5.12
TARGET-10-PAPJHR	M	11.00	B-Precursor	1	(-)	NA	(-)	NA	NA	NA	Dead	3.25
TARGET-10-PAPJIB	F	2.47	B-Precursor	2	(-)	(-)	(-)	(-)	(-)	53	Dead	3.68
TARGET-10-PAPJXI	F	8.90	B-Precursor	1	(-)	(-)	(-)	(-)	(-)	45	Alive	8.84
TARGET-10-PAPKNJ	F	4.32	B-Precursor	1	(-)	NA	(-)	(-)	(-)	47	Alive	9.52
TARGET-10-PAPLTZ	M	5.56	B-Precursor	1	(+)	(-)	(-)	(-)	(-)	51	Dead	7.08
TARGET-10-PAPLUG	M	1.39	B-Precursor	2	(-)	NA	(-)	NA	NA	NA	Dead	3.16
TARGET-10-PAPMVB	M	1.91	B-Precursor	1	(+)	(-)	(-)	(-)	(-)	47	Dead	3.87
TARGET-10-PAPNMY	M	13.99	B-Precursor	1	(-)	(-)	(-)	(-)	(-)	46	Dead	5.39
TARGET-10-PAPRCS	M	14.09	B-Precursor	2	(-)	(-)	(-)	(-)	(-)	46	Dead	1.26
TARGET-10-PAPRFE	F	2.57	B-Precursor	1	(-)	(+)	(-)	(-)	(-)	46	Dead	1.07
TARGET-10-PAPRMM	M	3.76	B-Precursor	2	(-)	NA	(-)	(-)	(-)	46	Dead	0.32
TARGET-10-PAPSPN	M	11.43	B-Precursor	1	(-)	(-)	(-)	(-)	(-)	46	Dead	2.01
TARGET-10-PAPTAT	M	2.54	B-Precursor	1	(-)	NA	(-)	(-)	(-)	46	Alive	8.93
TARGET-10-PAPTLM	F	1.86	B-Precursor	2	(-)	(-)	(-)	(-)	(-)	46	Dead	2.52

TARGET-10-PAPVTA	M	2.79	B-Precursor	1	(-)	(-)	(-)	(-)	(-)	51	Alive	8.81
TARGET-10-PAPZNK	F	10.02	B-Precursor	1	(-)	NA	(-)	(-)	(-)	NA	Dead	2.42
TARGET-10-PARACA	M	2.66	B-Precursor	2	(-)	(+)	(-)	(-)	(-)	46	Dead	2.21
TARGET-10-PARAKF	F	8.35	B-Precursor	1	(+)	NA	(-)	(-)	(-)	NA	Alive	5.32
TARGET-10-PARAPE	F	3.22	B-Precursor	1	(-)	NA	(-)	(-)	(-)	46	Alive	8.19
TARGET-10-PARARJ	F	3.09	B-Precursor	1	(-)	NA	(-)	(-)	(-)	NA	Dead	1.25
TARGET-10-PARBGL	F	3.25	B-Precursor	1	(-)	NA	(-)	(-)	(-)	47	Alive	8.89
TARGET-10-PARBRK	F	7.22	B-Precursor	1	(-)	NA	(-)	NA	(-)	NA	Dead	0.27
TARGET-10-PARBVI	F	13.55	B-Precursor	1	(-)	(-)	(-)	(-)	(-)	46	Dead	3.21
TARGET-10-PARDWN	F	1.97	B-Precursor	1	(-)	NA	(-)	(-)	(-)	45	Alive	8.24
TARGET-10-PARFTR	M	5.82	B-Precursor	1	(-)	(+)	(-)	(-)	(-)	46	Dead	1.27
TARGET-10-PARJLA	M	9.91	B-Precursor	2	(-)	NA	(-)	(-)	(-)	45	Alive	7.97
TARGET-10-PARJSR	M	11.21	B-Precursor	1	(-)	(-)	(-)	(-)	(-)	55	Dead	2.34
TARGET-10-PARJZZ	M	4.21	B-Precursor	1	(-)	(-)	(-)	(-)	(-)	47	Dead	2.72
TARGET-10-PARLAF	M	3.97	B-Precursor	1	(+)	(-)	(-)	(-)	(+)	89	Alive	8.05
TARGET-10-PARPNM	F	13.53	B-Precursor	1	(-)	(-)	(-)	(-)	(-)	46	Dead	0.64
TARGET-10-PARSLL	F	4.52	B-Precursor	2	(-)	NA	(-)	(-)	(-)	45	Alive	6.93
TARGET-10-PARSZH	F	12.92	B-Precursor	1	(-)	NA	(-)	(-)	(-)	NA	Alive	6.57
TARGET-10-PARTJL	F	2.90	B-Precursor	1	(-)	NA	(-)	(-)	(-)	NA	Alive	2.65
TARGET-10-PARTKL	F	2.98	B-Precursor	1	(-)	(-)	(-)	(-)	(-)	51	Dead	2.46
TARGET-10-PARUAT	F	2.50	B-Precursor	1	(-)	NA	(-)	(-)	(-)	45	Alive	8.11
TARGET-10-PARUFL	F	4.59	B-Precursor	1	(-)	NA	(-)	(-)	(-)	48	Alive	7.83
TARGET-10-PARUGP	M	2.08	B-Precursor	1	(-)	(+)	(-)	(-)	(-)	NA	Dead	0.79
TARGET-10-PARUNW	F	1.38	B-Precursor	2	(-)	NA	(-)	(-)	(-)	45	Alive	7.64
TARGET-10-PARWRJ	F	1.05	B-Precursor	2	(-)	NA	(-)	(-)	(-)	45	Alive	7.98
TARGET-10-PARXCD	F	14.37	B-Precursor	2	(-)	NA	(-)	(-)	(-)	45	Alive	3.63
TARGET-10-PARXMC	F	6.92	B-Precursor	1	(+)	NA	(-)	(-)	(-)	NA	Alive	7.72

TARGET-10-PARYAJ	M	1.16	B-Precursor	1	(-)	NA	(-)	(-)	(-)	45	Dead	7.38
TARGET-10-PARZYX	M	11.40	B-Precursor	1	(-)	NA	(-)	(-)	(-)	45	Alive	7.57
TARGET-10-PASEVJ	F	11.19	B-Precursor	1	(-)	NA	(-)	(-)	(-)	46	Dead	2.44
TARGET-10-PASHUI	M	5.11	B-Precursor	2	(-)	NA	(-)	(-)	(-)	NA	Dead	1.66
TARGET-10-PASILP	M	9.93	B-Precursor	2	(-)	NA	(-)	(-)	(-)	46	Dead	2.90
TARGET-10-PASKAY	M	1.60	B-Precursor	1	(-)	NA	(-)	(-)	(+)	NA	Dead	1.33
TARGET-10-PASMGZ	F	4.51	B-Precursor	1	(-)	NA	(-)	(-)	(-)	49	Alive	6.58
TARGET-10-PASNJI	M	3.75	B-Precursor	1	(-)	NA	(-)	(-)	(-)	46	Alive	6.09
TARGET-10-PASREU	F	1.87	B-Precursor	1	(-)	NA	(-)	(-)	(-)	47	Alive	6.58
TARGET-10-PASTSR	M	10.44	B-Precursor	1	(-)	NA	(-)	(-)	(-)	45	Alive	6.80
TARGET-10-PASTYT	M	3.03	B-Precursor	1	(-)	NA	(-)	(-)	(-)	46	Alive	6.66
TARGET-10-PASUBW	M	13.96	B-Precursor	1	(-)	(-)	(-)	(-)	(-)	48	Dead	1.30
TARGET-10-PASXFY	F	2.60	B-Precursor	1	(-)	NA	(-)	(-)	(-)	45	Alive	5.96
TARGET-10-PASXZH	M	3.24	B-Precursor	1	(-)	NA	(-)	(-)	(-)	46	Alive	6.15
TARGET-10-PATCJJ	F	10.34	B-Precursor	1	(-)	NA	(-)	(-)	(-)	45	Alive	6.29
TARGET-10-PATCTI	M	6.61	B-Precursor	1	(-)	(+)	(-)	(-)	(-)	46	Dead	1.32
TARGET-10-PATEVE	M	7.55	B-Precursor	1	(-)	NA	(-)	(-)	(-)	46	Alive	5.89

Table S3. Comparison of clinical characteristics according to AL133346.1 and CCN2 expression in pediatric B-ALL patients from TARGET dataset. Chr: Chromosome; NA: not available.

	**Pediatric B-ALL TCGA Population (N = 120)**							
	**AL133346.1 Expression**				**CCN2 Expression**			
	**Downexpression (N = 60)**	**OverExpression (N = 60)**	**p-value**	**p-value adj (FDR)**	**Downexpression (N = 60)**	**Overexpression (N = 60)**	**p-value**	**p-value adj (FDR)**
**Sex**								
Female	30 (50.0%)	31 (51.7%)	1	1	33 (55.0%)	28 (46.7%)	0.465	0.465
Male	30 (50.0%)	29 (48.3%)			27 (45.0%)	32 (53.3%)		

**Age at Diagnosis (years)**								
1-9.99	47 (78.3%)	44 (73.3%)	0.670	0.984	41 (68.3%)	50 (83.3%)	0.087	0.174
≥ 10	13 (21.7%)	16 (26.7%)			19 (31.7%)	10 (16.7%)		
**CNS**								
1	48 (80.0%)	45 (75.0%)	0.738	0.984	47 (78.3%)	46 (76.7%)	0.282	0.367
2	11 (18.3%)	13 (21.7%)			13 (21.7%)	11 (18.3%)		
3	1 (1.7%)	2 (3.3%)			0 (0%)	3 (5.0%)		
**t(12;21): ETV6-RUNX1 Fusion**								
Negative	54 (98.2%)	37 (80.4%)	0.005	0.020	57 (100%)	34 (77.3%)	0.0001	0.0008
Positive	1 (1.8%)	9 (19.6%)			0 (0%)	10 (22.7%)		
**t(1;19): TCF3-PBX1 Fusion**								
Negative	24 (82.8%)	43 (95.6%)	0.103	0.275	26 (78.8%)	41 (100%)	0.002	0.008
Positive	5 (17.2%)	2 (4.4%)			7 (21.2%)	0 (0%)		
**t(9;22): BCR-ABL1 Fusion**								
Negative	59 (98.3%)	58 (96.7%)	1	1	60 (100%)	57 (95.0%)	0.244	0.367
Positive	1 (1.7%)	2 (3.3%)			0 (0%)	3 (5.0%)		
**MLL Rearrangements - Status**								
Negative	57 (100%)	53 (100%)	NA	NA	56 (100%)	54 (100%)	NA	NA
Positive	0 (0%)	0 (0%)			0 (0%)	0 (0%)		
**Trisomy 4 and 10 - Status**								
Positive	54 (94.7%)	48 (87.3%)	0.199	0.398	53 (94.6%)	49 (87.5%)	0.321	0.367
Negative	3 (5.3%)	7 (12.7%)			3 (5.4%)	7 (12.5%)		

**Ploidy**								
Hipodiploidy (< 46 Chr)	16 (29.6%)	1 (2.1%)	0.0002	0.002	12 (24.5%)	5 (9.6%)	0.05	0.133
Diploidy (46 Chr)	24 (44.4%)	21 (44.7%)			23 (46.9%)	22 (42.3%)		
Partial Hyperdiploidy (47-50 Chr)	9 (16.7%)	10 (21.3%)			9 (18.4%)	10 (19.2%)		
High Hyperdiploidy (≥ 51)	5 (9.3%)	15 (31.9%)			5 (10.2%)	15 (28.9%)		

Table S4. Cox Univariate and multivariate overall analysis of clinical covariates in pediatric B-ALL patients from TARGET dataset. NA: Not included in the multivariate analysis. The "MLL Rearrangements – Status" covariate was excluded from the analysis because it did not have positive samples.

	**Univariate Cox Analysis**		**Multivariate Cox Analysis**	
	**Hazard Ratio (95% CI)**	**p-value**	**Hazard Ratio (95% CI)**	**p-value**
**Sex (Male vs Female)**	1.852 (1.062-3.228)	0.030	1.900 (0.908-3.973)	0.088
**Age at Diagnosis (≥ 10 vs 1-9.9 years)**	1.500 (0.823-2.733)	0.186	1.140 (0.510-2.550)	0.750
**CNS (2-3 vs 1)**	1.024 (0.538-1.949)	0.943	NA	NA
**t(12;21): ETV6-RUNX1 Fusion (Positive vs Negative)**	0.697 (0.250-1.944)	0.490	NA	NA
**t(1;19): TCF3-PBX1 Fusion (Positive vs Negative)**	21.840 (6.968-68.480)	1.22E-07	7.589 (1.914-30.085)	0.003
**t(9;22): BCR-ABL1 Fusion (Positive vs Negative)**	0.667 (0.092-4.834)	0.689	NA	NA
**Trisomy 4 and 10 - Status (Positive vs Negative)**	0.343 (0.192-1.986)	0.418	NA	NA
**Ploidy (Hipodiploidy vs Diploidy)**	0.126 (0.030-0.531)	0.005	0.237 (0.030-1.881)	0.173
**Ploidy (Partial Hyperdiploidy vs Diploidy)**	0.383 (0.158-0.926)	0.033	0.650 (0.252-1.679)	0.374
**Ploidy (High Hyperdiploidy vs Diploidy)**	0.346 (0.143-0.838)	0.019	0.386(0.148-1.006)	0.051
**CCN2 Expression (High vs Low)**	0.566 (0.328-0.980)	0.042	0.448 (0.204-0.984)	0.045
**AL133346.1 Expression (High vs Low)**	1.084 (0.632-1.859)	0.770	NA	NA

Table S5. Demographic and clinical characteristics of the pediatric T-ALL patients of the TARGET dataset. Abbreviations: CNS: central nervous system; CNS 1 means no CNS involvement, CNS 2 is a very low level, and CNS 3 is definite CNS involvement. F: female; M: male; NA: not available; MLL: MLL gene; OS: Overall Survival; t(12;21): translocation(12;21); t(1;19): translocation(1;19); t(9;22): translocation(9;22); (+): Positive; (-): Negative.

Sample	Sex	Age at Diagnosis (years)	Phenotype	CNS	t(12;21): ETV6-RUNX1 Fusion	t(1;19): TCF3-PBX1 Fusion	t(9;22): BCR-ABL1 Fusion	MLL Rearrangements - Status	Trisomy 4 and 10 - Status	№ Chromosomes	Vital Status	OS Time (years)
TARGET-10-PARASZ	F	4.41	T Cell ALL	2	(+)	(+)	(+)	(+)	(+)	46	Alive	8.45
TARGET-10-PAREGZ	F	8.56	T Cell ALL	1	NA	(+)	(+)	(+)	(+)	46	Alive	8.45
TARGET-10-PARFIH	M	5.27	T Cell ALL	1	NA	(+)	(+)	(+)	(+)	46	Alive	3.01
TARGET-10-PARFPJ	M	3.69	T Cell ALL	2	NA	NA	(+)	NA	NA	46	Alive	8.07
TARGET-10-PARFXJ	M	11.62	T Cell ALL	1	(+)	(+)	(+)	(+)	(+)	46	Alive	8.18
TARGET-10-PARGFL	M	4.10	T Cell ALL	3	NA	(+)	(+)	(+)	(+)	52	Dead	5.18
TARGET-10-PARHBI	M	7.89	T Cell ALL	1	(+)	NA	(+)	(+)	(+)	46	Alive	8.24
TARGET-10-PARHES	M	4.65	T Cell ALL	1	NA	NA	(+)	(+)	NA	NA	Alive	8.05
TARGET-10-PARIYD	M	8.97	T Cell ALL	1	NA	NA	(+)	(+)	NA	46	Alive	8.51
TARGET-10-PARIZN	M	10.20	T Cell ALL	3	NA	NA	(+)	(+)	NA	46	Dead	1.84
TARGET-10-PARJNX	F	3.10	T Cell ALL	2	(+)	(+)	(+)	(+)	(+)	46	Alive	8.23
TARGET-10-PARJPL	M	3.76	T Cell ALL	1	NA	(+)	(+)	(+)	(+)	46	Alive	8.65
TARGET-10-PARJXW	M	14.82	T Cell ALL	1	NA	(+)	(+)	(+)	(+)	46	Alive	0.10
TARGET-10-PARKLK	M	1.81	T Cell ALL	1	(+)	NA	(+)	(+)	(+)	46	Alive	0.08
TARGET-10-PARLJA	M	8.63	T Cell ALL	2	(+)	(+)	(+)	(+)	(+)	46	Alive	8.03
TARGET-10-PARLST	M	9.44	T Cell ALL	2	(+)	NA	(+)	(+)	(+)	NA	Alive	8.37
TARGET-10-PARMFF	M	8.32	T Cell ALL	1	(+)	(+)	(+)	(+)	(+)	44	Alive	8.73
TARGET-10-PARMIH	M	12.42	T Cell ALL	1	NA	(+)	(+)	(+)	(+)	46	Dead	8.08
TARGET-10-PARMKK	M	3.35	T Cell ALL	2	NA	(+)	(+)	(+)	(+)	46	Alive	8.22

TARGET-10-PARMMV	M	6.36	T Cell ALL	1	NA	NA	(+)	(+)	NA	46	Alive	8.36
TARGET-10-PARMRF	M	5.98	T Cell ALL	NA	NA	NA	(+)	NA	NA	46	Alive	8.18
TARGET-10-PARNBN	M	10.06	T Cell ALL	1	NA	(+)	(+)	(+)	(+)	46	Alive	4.62
TARGET-10-PARNEH	M	4.01	T Cell ALL	1	(+)	(+)	(+)	(+)	(+)	46	Dead	1.43
TARGET-10-PARNMV	M	9.53	T Cell ALL	1	NA	(+)	(+)	(+)	(+)	46	Alive	7.95
TARGET-10-PARNSP	M	6.04	T Cell ALL	1	(+)	(+)	(+)	(+)	(+)	47	Alive	8.45
TARGET-10-PARNXJ	M	4.82	T Cell ALL	1	NA	(+)	(+)	(+)	(+)	46	Alive	7.78
TARGET-10-PARPET	F	4.96	T Cell ALL	1	(+)	NA	(+)	(+)	(+)	46	Alive	5.31
TARGET-10-PARPHB	M	1.10	T Cell ALL	3	NA	(+)	(+)	(+)	(+)	46	Alive	8.10
TARGET-10-PARPUL	M	2.25	T Cell ALL	1	(+)	(+)	(+)	(+)	(+)	46	Alive	7.73
TARGET-10-PARPYJ	F	7.44	T Cell ALL	2	NA	(+)	(+)	(+)	(+)	46	Alive	7.55
TARGET-10-PARRKK	M	8.88	T Cell ALL	1	(+)	(+)	(+)	(+)	(+)	46	Alive	7.53
TARGET-10-PARSET	M	10.91	T Cell ALL	1	NA	NA	(+)	NA	NA	NA	Alive	0.97
TARGET-10-PARSJG	M	11.97	T Cell ALL	1	NA	(+)	(+)	(+)	(+)	46	Alive	7.49
TARGET-10-PARSNX	M	13.46	T Cell ALL	2	NA	(+)	(+)	(+)	(+)	46	Alive	5.69
TARGET-10-PARTBP	M	5.63	T Cell ALL	1	NA	(+)	(+)	(+)	(+)	46	Alive	8.44
TARGET-10-PARTLY	M	12.30	T Cell ALL	1	NA	(+)	(+)	(+)	(+)	49	Alive	8.37
TARGET-10-PARUEU	M	5.57	T Cell ALL	1	(+)	(+)	(+)	(+)	(+)	46	Alive	6.15
TARGET-10-PARUKW	F	9.79	T Cell ALL	1	(+)	(+)	(+)	(+)	(+)	46	Alive	7.39
TARGET-10-PARVEI	F	3.43	T Cell ALL	1	(+)	(+)	(+)	(+)	(+)	46	Alive	7.58
TARGET-10-PARVMR	M	4.96	T Cell ALL	1	NA	(+)	(+)	(+)	(+)	46	Alive	7.78

TARGET-10-PARWLP	M	7.90	T Cell ALL	2	NA	NA	(+)	(+)	NA	NA	Alive	7.90
TARGET-10-PARWMF	M	11.70	T Cell ALL	1	(+)	(+)	(+)	(+)	(+)	46	Alive	7.30
TARGET-10-PARWNW	M	11.95	T Cell ALL	1	NA	(+)	(+)	(+)	(+)	46	Alive	6.36
TARGET-10-PARXLS	M	4.38	T Cell ALL	1	(+)	(+)	(+)	(+)	(+)	46	Alive	7.55
TARGET-10-PARXVS	M	11.75	T Cell ALL	1	NA	(+)	(+)	(+)	(+)	46	Dead	6.27
TARGET-10-PASFHR	M	14.57	T Cell ALL	1	NA	NA	(+)	NA	NA	46	Alive	7.21
TARGET-10-PASFKA	M	12.77	T Cell ALL	1	(+)	NA	(+)	(+)	(+)	46	Alive	7.14
TARGET-10-PASFLK	M	12.02	T Cell ALL	1	(+)	NA	(+)	(+)	(+)	NA	Alive	7.32
TARGET-10-PASHDV	M	11.66	T Cell ALL	2	NA	(+)	(+)	(+)	(+)	46	Alive	7.31
TARGET-10-PASHNK	M	6.61	T Cell ALL	1	(+)	NA	(+)	(+)	(+)	46	Alive	7.06
TARGET-10-PASHUP	M	9.68	T Cell ALL	2	(+)	(+)	(+)	(+)	(+)	46	Alive	6.69
TARGET-10-PASHXL	F	13.53	T Cell ALL	1	NA	NA	(+)	NA	NA	NA	Alive	6.82
TARGET-10-PASIIY	M	5.94	T Cell ALL	1	NA	NA	(+)	NA	NA	46	Alive	3.75
TARGET-10-PASILW	F	12.42	T Cell ALL	2	(+)	(+)	(+)	(+)	(+)	46	Dead	4.56
TARGET-10-PASINX	M	11.36	T Cell ALL	1	NA	(+)	(+)	(+)	(+)	46	Alive	6.89
TARGET-10-PASJJR	M	14.28	T Cell ALL	1	(+)	(+)	(+)	(+)	(+)	46	Alive	6.85
TARGET-10-PASJLN	M	10.93	T Cell ALL	1	NA	(+)	(+)	(+)	(+)	46	Alive	7.29
TARGET-10-PASJMK	F	6.30	T Cell ALL	2	NA	NA	(+)	NA	NA	46	Alive	7.24
TARGET-10-PASJYI	M	14.31	T Cell ALL	1	(+)	NA	(+)	(+)	(+)	46	Alive	6.68
TARGET-10-PASKAD	F	10.43	T Cell ALL	3	NA	(+)	(+)	(+)	(+)	46	Alive	6.94
TARGET-10-PASKCL	M	12.68	T Cell ALL	2	(+)	NA	(+)	(+)	(+)	NA	Alive	6.95
TARGET-10-PASKIC	M	11.20	T Cell ALL	1	(+)	NA	(+)	(+)	(+)	NA	Alive	7.42
TARGET-10-PASKSY	M	9.72	T Cell ALL	1	NA	(+)	(+)	(+)	(+)	46	Alive	6.48

TARGET-10-PASKTG	M	5.83	T Cell ALL	1	NA	NA	(+)	NA	NA	NA	Alive	5.83
TARGET-10-PASKXN	M	9.42	T Cell ALL	1	(+)	(+)	(+)	(+)	(+)	46	Alive	1.59
TARGET-10-PASLAB	F	4.81	T Cell ALL	1	NA	(+)	(+)	(+)	(+)	46	Alive	6.89
TARGET-10-PASLBB	M	7.87	T Cell ALL	1	(+)	NA	(+)	(+)	(+)	NA	Alive	6.78
TARGET-10-PASMHF	F	12.07	T Cell ALL	3	(+)	(+)	(+)	(+)	(+)	46	Alive	6.93
TARGET-10-PASMIC	F	13.46	T Cell ALL	2	NA	(+)	(+)	(+)	(+)	46	Alive	6.84
TARGET-10-PASMNV	M	12.76	T Cell ALL	1	NA	NA	(+)	NA	NA	46	Alive	6.83
TARGET-10-PASNEH	M	5.93	T Cell ALL	1	NA	(+)	(+)	(+)	(+)	45	Alive	7.06
TARGET-10-PASNTZ	M	7.19	T Cell ALL	1	NA	NA	(+)	NA	NA	46	Alive	6.81
TARGET-10-PASPDS	M	10.92	T Cell ALL	2	NA	NA	(+)	NA	NA	46	Alive	7.19
TARGET-10-PASPPN	M	12.57	T Cell ALL	2	(+)	(+)	(+)	(+)	(+)	46	Dead	0.69
TARGET-10-PASRCV	F	3.40	T Cell ALL	1	NA	NA	(+)	(+)	NA	46	Alive	6.71
TARGET-10-PASRMM	M	4.37	T Cell ALL	1	NA	(+)	(+)	(+)	(+)	46	Alive	6.80
TARGET-10-PASSEF	F	4.94	T Cell ALL	2	NA	(+)	(+)	(+)	(+)	46	Alive	6.81
TARGET-10-PASSPP	M	9.97	T Cell ALL	2	NA	(+)	(+)	(+)	(+)	46	Alive	6.53
TARGET-10-PASSRP	M	4.50	T Cell ALL	1	NA	NA	(+)	(+)	NA	NA	Alive	3.37
TARGET-10-PASSSR	M	7.88	T Cell ALL	1	NA	NA	(+)	NA	NA	46	Alive	6.74
TARGET-10-PASSZA	M	14.83	T Cell ALL	1	(+)	(+)	(+)	(+)	(+)	46	Alive	6.83
TARGET-10-PASTDU	M	8.86	T Cell ALL	1	(+)	NA	(+)	(+)	(+)	NA	Alive	6.86
TARGET-10-PASTHE	F	6.37	T Cell ALL	1	NA	(+)	(+)	(+)	(+)	47	Alive	6.96
TARGET-10-PASTLP	M	9.36	T Cell ALL	1	NA	(+)	(+)	(+)	(+)	46	Alive	6.13
TARGET-10-PASTPT	F	4.86	T Cell ALL	1	(+)	(+)	(+)	(+)	(+)	49	Alive	6.82
TARGET-10-PASTXU	F	12.90	T Cell ALL	2	NA	NA	(+)	NA	NA	46	Alive	5.48

TARGET-10-PASUGC	M	9.62	T Cell ALL	2	NA	(+)	(+)	(+)	(+)	46	Alive	6.60
TARGET-10-PASUIN	M	13.70	T Cell ALL	2	NA	(+)	(+)	(+)	(+)	46	Alive	7.02
TARGET-10-PASUSV	M	8.56	T Cell ALL	1	NA	(+)	(+)	(+)	(+)	46	Alive	6.99
TARGET-10-PASVIN	M	7.17	T Cell ALL	1	NA	(+)	(+)	(+)	(+)	46	Alive	6.65
TARGET-10-PASVPZ	M	3.65	T Cell ALL	1	(+)	NA	(+)	(+)	(+)	NA	Alive	6.66
TARGET-10-PASWFN	M	11.32	T Cell ALL	1	(+)	NA	(+)	(+)	(+)	46	Alive	6.56
TARGET-10-PASWSR	F	3.42	T Cell ALL	1	NA	(+)	(+)	(+)	(+)	46	Alive	6.34
TARGET-10-PASWXB	M	9.65	T Cell ALL	3	NA	NA	(+)	NA	NA	46	Alive	6.26
TARGET-10-PASWXZ	F	7.55	T Cell ALL	1	(+)	(+)	(+)	(+)	(+)	46	Alive	6.68
TARGET-10-PASWZJ	M	5.22	T Cell ALL	2	NA	NA	(+)	NA	NA	46	Alive	6.58
TARGET-10-PASXIL	M	8.82	T Cell ALL	3	NA	(+)	(+)	(+)	(+)	46	Alive	6.83
TARGET-10-PASXLT	M	4.08	T Cell ALL	1	NA	(+)	(+)	(+)	(+)	46	Alive	6.62
TARGET-10-PASXLZ	M	5.23	T Cell ALL	1	NA	NA	(+)	NA	NA	46	Alive	6.33
TARGET-10-PASXMF	M	10.44	T Cell ALL	2	NA	NA	(+)	NA	NA	NA	Alive	6.46
TARGET-10-PASXSI	M	8.96	T Cell ALL	3	NA	(+)	(+)	(+)	(+)	46	Alive	6.63
TARGET-10-PASXUC	F	6.82	T Cell ALL	1	NA	(+)	(+)	(+)	(+)	89	Alive	6.13
TARGET-10-PASXUU	M	7.01	T Cell ALL	1	NA	(+)	(+)	(+)	(+)	46	Alive	6.31
TARGET-10-PASYAJ	M	3.05	T Cell ALL	1	NA	(+)	(+)	(+)	(+)	46	Alive	6.64
TARGET-10-PASYCN	M	10.98	T Cell ALL	2	(+)	(+)	(+)	(+)	(+)	50	Alive	6.33
TARGET-10-PASYIS	F	8.04	T Cell ALL	2	(+)	(+)	(+)	(+)	(+)	45	Alive	6.26
TARGET-10-PASYSJ	M	14.73	T Cell ALL	3	(+)	(+)	(+)	(+)	(+)	46	Alive	5.82
TARGET-10-PASZEW	F	7.34	T Cell ALL	1	NA	(+)	(+)	(+)	(+)	46	Alive	6.34
TARGET-10-PASZIY	M	14.33	T Cell ALL	1	(+)	(+)	(+)	(+)	(+)	50	Alive	6.49
TARGET-10-PATALJ	M	12.78	T Cell ALL	1	NA	(+)	(+)	(+)	(+)	46	Alive	5.21
TARGET-10-PATAXS	M	1.00	T Cell ALL	1	(+)	NA	(+)	(+)	(+)	NA	Alive	6.17

TARGET-10-PATAYT	F	5.91	T Cell ALL	2	NA	(+)	(+)	(+)	(+)	46	Alive	6.49
TARGET-10-PATBDK	M	7.94	T Cell ALL	2	NA	NA	(+)	NA	NA	46	Alive	6.33
TARGET-10-PATBGC	F	10.80	T Cell ALL	1	NA	(+)	(+)	(+)	(+)	46	Alive	5.04
TARGET-10-PATBNT	M	13.52	T Cell ALL	2	(+)	(+)	(+)	(+)	(+)	46	Alive	5.90
TARGET-10-PATBRV	F	8.36	T Cell ALL	1	NA	(+)	(+)	(+)	(+)	46	Dead	0.58
TARGET-10-PATBTX	M	3.15	T Cell ALL	1	(+)	NA	(+)	(+)	(+)	46	Alive	5.98
TARGET-10-PATCDM	M	3.26	T Cell ALL	3	(+)	(+)	(+)	(+)	(+)	46	Dead	2.60
TARGET-10-PATCKV	M	14.42	T Cell ALL	1	NA	(+)	(+)	(+)	(+)	50	Alive	6.59
TARGET-10-PATCNI	F	4.61	T Cell ALL	1	(+)	(+)	(+)	(+)	(+)	46	Alive	6.57
TARGET-10-PATCUK	M	12.04	T Cell ALL	1	(+)	(+)	(+)	(+)	(+)	46	Alive	6.14
TARGET-10-PATCZN	F	4.94	T Cell ALL	1	NA	NA	(+)	NA	NA	46	Alive	6.30
TARGET-10-PATDBU	M	9.72	T Cell ALL	2	NA	(+)	(+)	(+)	(+)	46	Alive	6.39
TARGET-10-PATDDZ	M	12.59	T Cell ALL	1	(+)	(+)	(+)	(+)	(+)	46	Alive	5.98
TARGET-10-PATDFE	F	5.02	T Cell ALL	1	NA	NA	(+)	(+)	NA	46	Alive	6.48
TARGET-10-PATDGZ	M	2.57	T Cell ALL	1	(+)	(+)	(+)	(+)	(+)	46	Alive	6.36
TARGET-10-PATDLG	M	11.79	T Cell ALL	1	(+)	(+)	(+)	(+)	(+)	46	Alive	6.44
TARGET-10-PATDMN	M	6.92	T Cell ALL	1	NA	NA	(+)	NA	NA	46	Alive	6.36
TARGET-10-PATEFF	M	6.48	T Cell ALL	3	NA	(+)	(+)	(+)	(+)	46	Alive	5.79
TARGET-10-PATEIT	F	9.54	T Cell ALL	2	(+)	(+)	(+)	(+)	(+)	46	Dead	1.66
TARGET-10-PATENL	M	3.68	T Cell ALL	1	NA	(+)	(+)	(+)	(+)	46	Dead	1.66
TARGET-10-PATEVL	M	3.36	T Cell ALL	2	NA	(+)	(+)	(+)	(+)	46	Alive	6.40
TARGET-10-PATEYS	M	5.23	T Cell ALL	3	(+)	NA	(+)	(+)	(+)	46	Alive	5.29
TARGET-10-PATFJD	F	2.52	T Cell ALL	1	NA	(+)	(+)	(+)	(+)	47	Alive	6.28

TARGET-10-PATFJP	M	8.90	T Cell ALL	1	(+)	(+)	(+)	(+)	(+)	47	Alive	5.54
TARGET-10-PATFVG	M	5.83	T Cell ALL	1	NA	NA	(+)	NA	NA	46	Alive	6.00
TARGET-10-PATGKE	M	11.50	T Cell ALL	1	NA	NA	(+)	NA	NA	46	Alive	5.49
TARGET-10-PATGLV	M	6.18	T Cell ALL	1	(+)	(+)	(+)	(+)	(+)	46	Alive	6.27
TARGET-10-PATGMP	M	10.74	T Cell ALL	1	(+)	(+)	(+)	(+)	(+)	46	Alive	5.97
TARGET-10-PATGVX	M	7.30	T Cell ALL	1	(+)	(+)	(+)	(+)	(+)	46	Alive	6.31
TARGET-10-PATGWP	M	14.21	T Cell ALL	1	NA	(+)	(+)	(+)	(+)	45	Alive	6.03
TARGET-10-PATGXS	F	13.89	T Cell ALL	1	(+)	NA	(+)	(+)	(+)	NA	Alive	5.85
TARGET-10-PATGZA	F	14.38	T Cell ALL	2	NA	(+)	(+)	(+)	(+)	47	Alive	5.98
TARGET-10-PATHBG	F	2.96	T Cell ALL	1	(+)	(+)	(+)	(+)	(+)	46	Alive	5.96
TARGET-10-PATHFE	M	11.17	T Cell ALL	1	NA	(+)	(+)	(+)	(+)	46	Alive	5.96
TARGET-10-PATHGY	M	3.61	T Cell ALL	2	(+)	(+)	(+)	(+)	(+)	46	Alive	5.82
TARGET-10-PATHJF	F	10.25	T Cell ALL	1	(+)	NA	(+)	(+)	(+)	NA	Alive	5.58
TARGET-10-PATHRF	M	5.17	T Cell ALL	1	NA	NA	(+)	(+)	NA	NA	Alive	6.27
TARGET-10-PATHWV	M	8.56	T Cell ALL	2	(+)	(+)	(+)	(+)	(+)	46	Alive	6.17
TARGET-10-PATIBE	F	11.24	T Cell ALL	2	NA	(+)	(+)	(+)	(+)	47	Alive	5.82
TARGET-10-PATITY	M	8.56	T Cell ALL	2	NA	(+)	(+)	(+)	(+)	46	Alive	5.90
TARGET-10-PATJBC	M	5.33	T Cell ALL	1	NA	(+)	(+)	(+)	(+)	47	Alive	6.03
TARGET-10-PATJLT	M	12.09	T Cell ALL	3	(+)	(+)	(+)	(+)	(+)	46	Alive	6.03
TARGET-10-PATKGP	F	10.57	T Cell ALL	3	(+)	NA	(+)	(+)	(+)	46	Alive	5.93
TARGET-10-PATKVD	M	2.48	T Cell ALL	1	NA	NA	(+)	(+)	NA	NA	Alive	5.89

TARGET-10-PATKWU	M	13.23	T Cell ALL	2	(+)	(+)	(+)	(+)	(+)	46	Alive	5.70
TARGET-10-PATLGU	M	7.06	T Cell ALL	2	NA	NA	(+)	NA	NA	46	Alive	5.98
TARGET-10-PATLHH	M	7.75	T Cell ALL	1	(+)	(+)	(+)	(+)	(+)	46	Alive	5.92
TARGET-10-PATLHS	M	10.96	T Cell ALL	1	NA	NA	(+)	NA	NA	NA	Alive	4.25
TARGET-10-PATLNS	F	2.25	T Cell ALL	1	NA	(+)	(+)	(+)	(+)	46	Alive	5.71
TARGET-10-PATLNZ	M	9.86	T Cell ALL	1	NA	(+)	(+)	(+)	(+)	46	Alive	3.34
TARGET-10-PATLRZ	M	13.98	T Cell ALL	1	(+)	(+)	(+)	(+)	(+)	46	Alive	5.54
TARGET-10-PATMRE	M	6.77	T Cell ALL	1	(+)	(+)	(+)	(+)	(+)	46	Alive	5.75
TARGET-10-PATMTV	M	11.07	T Cell ALL	2	NA	(+)	(+)	(+)	(+)	46	Alive	5.97
TARGET-10-PATMXN	M	14.92	T Cell ALL	1	(+)	(+)	(+)	(+)	(+)	46	Alive	3.26
TARGET-10-PATMYZ	M	12.10	T Cell ALL	1	NA	(+)	(+)	(+)	(+)	46	Alive	5.77
TARGET-10-PATNAM	F	2.23	T Cell ALL	1	NA	(+)	(+)	(+)	(+)	46	Alive	5.90
TARGET-10-PATNIA	F	6.57	T Cell ALL	1	NA	(+)	(+)	(+)	(+)	46	Alive	5.89
TARGET-10-PATPDA	M	2.05	T Cell ALL	2	(+)	(+)	(+)	(+)	(+)	46	Alive	4.97
TARGET-10-PATRAB	F	4.67	T Cell ALL	2	NA	(+)	(+)	(+)	(+)	46	Alive	5.88
TARGET-10-PATRGV	F	1.58	T Cell ALL	3	NA	(+)	(+)	(+)	(+)	46	Dead	4.60
TARGET-10-PATRHL	M	6.29	T Cell ALL	1	NA	(+)	(+)	(+)	(+)	46	Alive	5.59
TARGET-10-PATRNA	M	2.20	T Cell ALL	2	NA	(+)	(+)	(+)	(+)	46	Alive	5.69
TARGET-10-PATRUN	M	4.83	T Cell ALL	1	NA	(+)	(+)	(+)	(+)	46	Alive	0.28
TARGET-10-PATRXL	M	4.84	T Cell ALL	2	(+)	NA	(+)	(+)	(+)	46	Alive	4.91
TARGET-10-PATSIY	M	9.32	T Cell ALL	1	NA	(+)	(+)	(+)	(+)	46	Alive	5.72
TARGET-10-PATSLH	M	2.26	T Cell ALL	1	NA	(+)	(+)	(+)	(+)	46	Alive	4.95

TARGET-10-PATWHB	F	10.69	T Cell ALL	3	NA	(+)	(+)	(+)	(+)	46	Alive	5.24
TARGET-10-PATWIJ	M	14.20	T Cell ALL	1	NA	(+)	(+)	(+)	(+)	92	Alive	5.31
TARGET-10-PATWJU	M	5.82	T Cell ALL	1	NA	(+)	(+)	(+)	(+)	47	Alive	5.51
TARGET-10-PATWYZ	M	3.79	T Cell ALL	1	NA	(+)	(+)	(+)	(+)	47	Alive	5.40
TARGET-10-PATXNK	M	9.39	T Cell ALL	1	NA	(+)	(+)	(+)	(+)	45	Alive	5.11
TARGET-10-PATXSK	M	6.24	T Cell ALL	2	NA	(+)	(+)	(+)	(+)	48	Alive	5.45
TARGET-10-PATYCH	M	3.92	T Cell ALL	1	(+)	NA	(+)	(+)	(+)	46	Alive	5.08
TARGET-10-PATYJK	M	12.19	T Cell ALL	1	NA	(+)	(+)	(+)	(+)	51	Alive	5.35
TARGET-10-PATYWV	M	7.66	T Cell ALL	1	NA	(+)	(+)	(+)	(+)	46	Dead	4.51
TARGET-10-PATZFF	F	10.69	T Cell ALL	1	NA	(+)	(+)	(+)	(+)	46	Alive	5.02
TARGET-10-PATZSL	F	10.03	T Cell ALL	1	(+)	(+)	(+)	(+)	(+)	46	Alive	5.19
TARGET-10-PATZVD	M	4.16	T Cell ALL	1	NA	NA	(+)	NA	NA	46	Alive	5.22
TARGET-10-PATZYC	M	3.70	T Cell ALL	1	(+)	(+)	(+)	(+)	(+)	46	Alive	5.15
TARGET-10-PAUACG	M	4.95	T Cell ALL	1	(+)	(+)	(+)	(+)	(+)	46	Alive	4.87
TARGET-10-PAUAFN	M	3.93	T Cell ALL	2	NA	(+)	(+)	(+)	(+)	46	Alive	5.08
TARGET-10-PAUAJA	M	11.89	T Cell ALL	1	(+)	(+)	(+)	(+)	(+)	46	Alive	5.13
TARGET-10-PAUAZV	M	8.32	T Cell ALL	1	NA	(+)	(+)	(+)	(+)	46	Alive	4.95
TARGET-10-PAUBCT	M	13.02	T Cell ALL	1	NA	(+)	(+)	(+)	(+)	46	Alive	5.05
TARGET-10-PAUBLL	F	7.16	T Cell ALL	1	NA	NA	(+)	NA	NA	NA	Alive	5.24
TARGET-10-PAUBPY	M	11.55	T Cell ALL	1	(+)	(+)	(+)	(+)	(+)	46	Alive	5.03
TARGET-10-PAUBTC	F	1.83	T Cell ALL	3	NA	NA	(+)	(+)	NA	NA	Alive	5.02
TARGET-10-PAUBXP	M	8.31	T Cell ALL	1	(+)	(+)	(+)	(+)	(+)	45	Alive	4.78
TARGET-10-PAUCDY	M	9.94	T Cell ALL	1	(+)	(+)	(+)	(+)	(+)	46	Alive	4.91
